# BRD4/nuclear PD-L1/RelB circuit is involved in the stemness of breast cancer cells

**DOI:** 10.1186/s12964-023-01319-6

**Published:** 2023-11-03

**Authors:** Su-Lim Kim, Hack Sun Choi, Dong-Sun Lee

**Affiliations:** 1 Bio-Health Materials Core-Facility Center, Jeju National University, Jeju, 63243 Republic of Korea; 2 Graduate Program for Bio-health/Innovative Drug Development using Subtropical Bio-Resources, Jeju National University, Jeju, 63243 Republic of Korea; 3 Subtropical/Tropical Organism Gene Bank, Jeju National University, Jeju, 63243 Republic of Korea; 4 Faculty of Biotechnology, College of Applied Life Sciences, Jeju National University, SARI, Jeju, 63243 Republic of Korea

**Keywords:** Breast cancer stem cell, BRD4, Nuclear PD-L1, RelB, IL-6

## Abstract

**Background:**

Breast cancer (BC) is the most common cancer diagnosed in women worldwide. BC stem cells (BCSCs) have been known to be involved in the carcinogenesis of the breast and contribute to therapeutic resistance. The programmed death-ligand 1 (PD-L1) expression of BC correlated with a poor prognosis. Immunotherapies that target PD-L1 have great potential and have been successful when applied to cancer treatment. However, whether PD-L1 regulates BCSC formation is unknown.

**Methods:**

BCSCs were enriched by serum-free suspension culture. The properties of BCSCs were examined by mammosphere formation assay, CD44^+^/Cd24^−^, aldehyde dehydrogenase (ALDH) assay, CSC marker analysis, and mammosphere growth assay. To elucidate the functions of bromodomain-containing protein 4 (BRD4), nuclear PD-L1, and RelB proteins in the stemness of BCSCs, mammosphere formation was examined using BRD4 inhibitor and degrader, PD-L1 degrader, and RelB inhibitor. The antitumor function of 3',4',7,8-tetrahydroxyflavone (THF), a specific BRD4 inhibitor, was studied through in vivo tumor model and mouse studies, and the protein levels of c-Myc, PD-L1, and RelB were examined in tumor model under THF treatment.

**Results:**

BRD4 was upregulated in breast CSCs and regulates the stemness of BCs. The downregulation of BRD4 using BRD4 PROTAC, ARV-825, and BRD4 inhibitor, (+)-JQ1, inhibits mammosphere formation and reduces the levels of breast CSC markers (CD44^+^/CD24^−^ and ALDH1), stem cell marker genes, and mammosphere growth. BRD4 inhibitor (JQ1) and degrader (ARV825) downregulate membrane and nuclear fractions of PD-L1 through the inhibition of PD-L1 transcript levels. The knockdown of PD-L1 inhibits mammosphere formation. Verteporfin, a PD-L1 degrader, inhibits the transcripts and protein levels of PD-L1 and downregulates the transcript and protein levels of RelB. Calcitriol, a RelB inhibitor, and the knockdown of RelB using si-RelB regulate mammosphere formation through interleukin-6 (IL-6) expression. THF is a natural product and a potent selective BRD4 inhibitor, inhibits mammosphere formation, and reduces the levels of CD44^+^/CD24^−^ and mammosphere growth by downregulating c-Myc, PD-L1, and RelB. 3',4',7,8-THF shows tumoricidal activity and increased levels of CD3^+^CD4^+^ and CD3^+^CD8^+^ T-cells in the tumor and tumor-draining lymph nodes (TDLNs) in the murine tumor model using 4T1 and MC38 cells.

**Conclusions:**

The results show the first evidence of the essential role of the BRD4/nuclear PD-L1/RelB axis in breast CSC formation. The nuclear PD-L1 regulates RelB, and the RelB/p65 complex induces IL6 and breast CSC formation. Targeting nuclear PD-L1 represents a potential and novel tool for immunotherapies of intractable BC.

Video Abstract

**Supplementary Information:**

The online version contains supplementary material available at 10.1186/s12964-023-01319-6.

## Background

Breast cancer (BC) is cancer that forms in normal breast tissues and can occur in men and women; however, it mainly occurs in women and threatens women’s health [1]. BC involves lobules, ducts, and connective tissue of the breast and has different physiological properties and clinical outcomes [2]. Breast cancer can be divided into luminal A, luminal B, HER2 overexpressing, and TNBC. TNBC accounts for 15–20% of BCs [3]. Patients with TNBC showed a poorer prognosis than patients with other BCs [4]. Despite advanced interventions, relapse and metastasis of BC reduced survival rates. These interventions are not the best option for the treatment of BC metastasis [5]. Understanding BC tumorigenesis-related signaling pathways might help in the development of a therapeutic approach for cancer treatment [2]. BC stem cells (BCSCs) are BC subpopulations and play an important role in the metastasis of BC and resistance to chemotherapy [6]. BCSCs are capable of self-renewal and differentiate into cancer cells. Much data show that BCSCs cause tumor progression and drug resistance to conventional therapy [7, 8]. Targeting breast CSCs may be a good tool for BC treatment [9–11].

BRD4 is a transcriptional and epigenetic factor that plays a vital role in embryogenesis and cancer development and is a member of the bromodomain and extraterminal domain (BET) family (BRD2, BRD3, and BRDT) [12]. Bromodomain-containing protein 4 (Brd4) supports tumor-driving oncogene expression. Since the BET family is a potential cancer therapeutic target, BET inhibitors are currently in preclinical and clinical trials for the treatment of multiple tumors [13]. BRD4 regulates the self-renewal ability of glioma-initiating cells by interaction in the Notch1 promoter region and involvement of tumor metabolism [14]. JQ1, BET (BRD2, BRD3, and BRD4) inhibitor, represents the anti-BCSC activity [15]. BRD4 regulates the transcription factor △Np63α to induce the CSC phenotype in squamous cell carcinoma [16]. However, whether the BRD4 signal is related to the formation of breast CSCs is unknown.

Programmed death-ligand 1 (PD-L1) is a cluster of differentiation 274 (CD274) or B7 homolog 1 (B7-H1) protein that is encoded by the *CD274* gene in humans [17]. The PD- programmed cell death protein 11/PD-L1 axis inhibits T-cell activation and cytotoxic secretion in tumors, is responsible for the cancer immune escape, and makes a huge effect on cancer therapy [18]. PD-L1 plays an important role in inhibiting immune response by modulating T-cell activation and inducing apoptosis of antigen-specific T-cells. PD-L1 attenuates the host immune response to tumor cells. Therefore, the PD-1/PD-L1 axis is responsible for cancer immune escape and gives bad effects on cancer therapy [18]. PD-L1 is overexpressed on breast CSCs through the notch3/mTOR axis and enhanced colorectal cancer stem cell (CSC) formation by activating the HMGA1-dependent pathway [19, 20]. Immune checkpoint Inhibitor therapy is one of the most promising anticancer therapies. Antibodies against the PD-1/PD-L1 axis have been applied to several cancers and have demonstrated good efficacy. Nevertheless, monotherapy with anti-PD-1/PD-L1 of metastatic BC showed a poor response [21]. The representative drug pembrolizumab contains an antibody that blocks PD-1 protein on the surface of T-cells, and chemotherapy as a first-line treatment is useful for the treatment of PD-L1-positive metastatic TNBC [22]. CSCs are not just resistant to chemotherapy but also immunotherapy. Whether the PL-L1 of CSCs is related to the formation of breast CSCs is unknown.

Transcription factors of the nuclear factor-kappa B (NF-κB) family regulating the immune responses have five members: c-Rel, p65 (RelA), RelB, p105/p50 (NF-κB1), and p100/p52 (NF-κB2) [23]. The NF-κB pathway activation plays an important role in the progression of BC, and levels of RelB protein are known to be very high in aggressive BC tissues, particularly in TNBC. RelB induced cell mobility and inhibited apoptosis of BC [24]. RelB increased the proliferation of human pluripotent stem cells (hPSCs) through IMP3- and LIN28-mediated regulation [25]. The constitutive activated NF-κB (p65/p50) has been seen in many tumor types and CSCs [26]. However, whether the RelB of CSCs is related to breast CSC formation is unknown.

In our study, we showed a novel molecular mechanism of breast CSC formation by BRD4/nuclear PD-L1/RelB axis in vitro and in vivo.

## Materials and methods

### Chemicals

The pan-BET inhibitor, (+)-JQ, and PD-L1 degrader, verteporfin, were purchased from Sigma-Aldrich (St. Louis, MO, USA). BRD4 degrader, ARV825, and RelB inhibitor, calcitriol, were purchased from MedChemExpress (Monmouth, NJ, USA). 3',4',7,8-tetrahydroxyflavone (THF), the BRD4 natural inhibitory compound, was purchased from Tocris Bioscience (Bristol, UK).

### Cell culture and culture conditions

MDA-MB-231, MCF-7 and HCC1937 cells were received from the Korea Cell Line Bank (Seoul, Republic of Korea). 4T1 cell were obtained from American Type Culture Collection (ATCC). MDA-MB-231 and HCC1937 cells were cultured in RPMI 1640 medium supplemented with 10% fetal bovine serum (FBS) and 1% penicillin/streptomycin (Thermo Fisher Scientific Inc., Waltham, MA, USA). 4T1 and MC38 cells were purchased from Kerafast Inc. (Boston, MA, USA). 4T1 cells were cultured in Dulbecco’s modified eagle’s medium supplemented with 10% fetal bovine serum (FBS) and 1% penicillin/streptomycin (Thermo Fisher Scientific Inc., USA). MC38 cells were cultured in Dulbecco’s modified eagle’s medium supplemented with 10% FBS, 2 mM glutamine, 0.1 mM nonessential amino acids, 1 mM sodium pyruvate, 10 mM Hepes, 25 µg/mL gentamycin sulfate, and 1% penicillin/streptomycin (Thermo Fisher Scientific Inc.). Cells were incubated at 37 °C in an atmosphere of 5% CO_2_. Cell culture ware, including cell culture dishes and plates, was purchased from SPL Life Sciences Co. (Pocheon-si, Gyeonggi-do, South Korea).

### CSC formation

For mammosphere formation, MCF-7, MDA-MB-231, 4T1 and HCC1937 cells (1 × 10^4^ cells /mL) were cultured in a cell floater plate containing MammoCult™ medium (STEMCELL Technologies, Vancouver, BC, CA) with heparin and hydrocortisone and incubated at 37 °C in an atmosphere of 5% CO_2_ for 7 days. For colon tumorsphere formation, MC38 cells (2 × 10^4^ cells/mL) were seeded in a cell floater plate containing Cancer Stem Premium Media (ProMab Biotechnologies Inc., Richmond, CA, USA) and incubated at 37 °C in an atmosphere of 5% CO_2_ for 5–7 days. To count CSCs, plates were scanned and analyzed using the NICE program. The CSC formation assay was determined by evaluating mammosphere formation efficiency (MFE) or tumorsphere formation efficiency (TFE) (%). Cell floater plates, including 6-well ultra-low attachment plates, were obtained from Corning (Corning, NY, USA) and SPL Life Sciences Co. (Pocheon-si, Gyeonggi-do, South Korea).

### Cell proliferation

MDA-MB-231 and HCC1937 cells were seeded in a 96-well plate and incubated for 24 h. The cells were treated with increasing concentrations of (+)-JQ1, ARV825, verteporfin, calcitriol, and THF for 24 h. The cell viability assay followed the manufacturer’s protocol of EZ-Cytox (Dogenbio, Seoul, South Korea). Then, 10 µL of EZ-Cytox solution was added per well. After incubation at 37 °C for 1 h, a VersaMax ELISA Microplate Reader (Molecular Devices, San Jose, CA, USA) was used for measurement at OD_450_.

### Target gene knockdown using small interfering RNA (siRNA)

To investigate the effects of BRD4, PD-L1, and RelB on mammosphere formation, MDA-MB-231 cells were transfected with specific siRNAs. siRNAs and scrambled siRNAs used in the study were obtained from Bioneer (Daejeon, South Korea). For the knockdown of target genes, cells were transfected using Lipofectamine 3000 (Invitrogen, Waltham, MA, USA) according to the manufacturer’s protocol. Whether knockdown succeeded by checking the levels of siRNA proteins through immunoblotting using target antibodies was determined.

### Transient transfection of pEGFP-N1/PD-L1 plasmid

MDA-MB-231 cells were cultured at 70% confluent on the day of transfection. Transient transfection was performed in a 6-well plate using Invitrogen™ Lipofectamine™ 3000 (Thermo Fisher Scientific, Waltham, MA, USA) and 2 µg of the PD-L1 expression plasmid, pEGFP-N1/PD-L1 (Addgene, Watertown, MA, USA). After transfection, The PD-L1 and GFP levels were determined using anti-PD-L1 and anti-GFP.

### RNA isolation and reverse-transcription quantitative polymerase chain reaction (RT-qPCR)

The total RNA was purified using MiniBEST Universal RNA Extraction Kit (Takara, Tokyo, Japan). RT-qPCR was performed using a TOPreal™ One-step RT-qPCR Kit (Enzynomics, Daejeon, South Korea). Our studies followed the manufacturer’s protocol. GAPDH primer was synthesized, and other primers (CD44, c-Myc, OCT4, SOX2, PD-L1, RelB, interleukin-6 [IL-6], and IL-8) were purchased in Bioneer. The *GAPDH* gene has been experimented with for use as an internal control. Primer sequences that were used to perform RT-qPCR are shown in Table 1.

**Table 1:** Primer sequences of target genes for reverse-transcription quantitative polymerase chain reaction

Gene name (human)	Primer sequences	
	Forward	Reverse
**GAPDH**	CACATGGCCTCCAAGGAGTAA	TGAGGGTCTCTCTCTTCCTCTTGT
**c-Myc**	AATGAAAGGCCCCCAAGGTAGTTATCC	AGCAAAACCCGGAGGAGT
**CD44**	AGAAGGTGTGGGCAGAAGAA	AAATGCACCATTTCCTGAGA
**OCT4**	AGCAAAACCCGGAGGAGT	CCACATCGGCTGTGTATATC
**SOX2**	TTGCTGCCTCTTTAAGACTAGGA	CTGGGGCTCAAACTTCTCTC
**CD274**	AAAGTCAATGCCCCATACGG	TTCTCTTCCCACTCACGGGT
**RelB**	GTCTTTCCCCACGAGGCTAT	CCGTACCTGGTCATCACAGAG
**IL-6**	ATGAACTCCTTCCTCCACAAGCGC	GAAGAGCCCTCAGGCTGGACTG
**IL-8**	CATACTCCAAACCTTTCCACCCC	TCAGCCCTCTTCAAAAACTTCTCCA

### Immunoblot and immunoprecipitation

Cancer cells and CSCs were treated with drugs, (+)-JQ1, ARV825, verteporfin, calcitriol, caffeic acid, and THF for 24 h, and cancer cells were centrifuged and lysed using RIPA buffer (Thermo Fisher Scientific) with protease inhibitors and phosphatase inhibitors. The fractionated proteins of cells were treated using a subcellular protein fractionation kit for cultured cells. The fractionated proteins of cells were isolated using a subcellular protein fractionation kit for cultured cells (Thermo Fisher Scientific). Lysates were separated by sodium dodecyl sulfate-polyacrylamide gel electrophoresis and electro-transferred to Immobilon®-FL polyvinylidene fluoride membranes (Millipore, Burlington, MA, USA). After that, we followed LI-COR (Lincoln, NE, USA) fluorescent Western blot detection protocol. The membrane was blocked with Odyssey® Blocking Buffer for 1 h at room temperature with gentle shaking. The primary antibody was added using the vendor’s recommendations, and the blot was incubated overnight at 4 °C. After the membranes were washed, they were incubated with fluorescent-labeled secondary antibodies for 1 h. The protein bands of the membranes were detected and quantitated using an Odyssey CLx imaging machine (LI-COR). To detect the interaction protein of RelB, we used Puredown Protein A/G-Agarose (GenDEPOT, Katy, TX, USA) for pulldown reactions. The beads were then washed with lysis buffer and finally eluted using lysis buffer followed by Western blot analysis. The antibodies used are as follows: anti-BRD4, anti-PD-L1, anti-RelB, anti-NF-κB, anti-c-Myc (Cell Signaling Technology; CST, Danvers, MA, USA), anti-α-tubulin, anti-vimentin, anti-β-actin (Santa Cruz Biotechnology, Dallas, TX, USA), anti-ATPase (Novus Biologicals, Minneapolis, MN, USA), anti-PD-L1 (R&D Systems, Minneapolis, MN, USA) and anti-Lamin-B (Invitrogen).

### Flow cytometry analysis

For detecting only BCSCs in cancers, cancer cells were stained with specific marker proteins using antibodies such as FITC anti-CD44 and APC anti-CD24 (BD, San Jose, CA, USA) to define CD44+/CD24-BCSCs. Single-cell suspensions were prepared from mouse tumor tissues or TDLNs to analyze the T-cells. The processes of tissue sample preparation are described in “Subcutaneous tumor and TDLN resection and sample preparation” section. Cells were stained with specific antibodies as APC anti-mouse CD8a, APC anti-mouse CD4, and FITC anti-mouse CD3 (BioLegend, San Diego, CA, USA) to define CD8+/CD3 + cytotoxic T-cells or CD4+/CD3 + helper T-cells. The samples were analyzed by flow cytometry (Accuri C6, BD Biosciences, East Rutherford, NJ, USA).

### ALDH detecting assay

ALDH detection was examined using an ALDEFUOR kit (STEMCELL Technologies). Assays were according to the vendor’s recommendation protocol. MDA-MB-231 cells, HCC1937 and MC 38 cells were cultured in 6-well plates and incubated for 24 h. The cells were incubated in ALDEFLUOR™ Reagent at 37 °C for 20 min. ALDH-positive cells were analyzed by performing a flow cytometer (Accuri C6, BD Biosciences). Single-cell suspensions were prepared from mouse tumor tissues or TDLNs to analyze CSCs. Samples containing diethylaminobenzaldehyde were used as negative controls. The processes of tissue sample preparation are described in “Subcutaneous tumor and TDLN resection and sample preparation” section. After sample preparation, the method is the same as described above.

### Luciferase assay

PD-L1-specific reporter plasmid, pGL3 1 kb promoter was received from Addgene (Watertown, MT, USA). Cancer cells were transfected with reporter plasmid using Lipofectamine 3000 (Invitrogen). PD-L1 reporter gene was analyzed with a luciferase assay system (Promega, Madison, WI, USA) and a luminometer. β-Galactosidase reporter gene was analyzed with Galacto-Light Plus™ β-Galactosidase Reporter Gene Assay System (Invitrogen) and has been experimented with for use as an internal control.

### Chromatin immunoprecipitation (ChIP) assay

To explore protein–DNA interactions, we used the cleavage under targets and release using nuclease method. To isolate the protein–DNA complex of interest, cells were harvested and bound to concanavalin A-coated magnetic beads. After binding, the membrane was permeabilized by the treatment of digitonin, and antibodies were bound to the transcription factor of interest. For DNA digestion, pAG-MNase fusion protein and Ca^2+^ were added. DNA was collected using DNA spin columns. The purified, enriched DNA was identified and quantitated by PCR using AccuPower® 2X GreenStar™ qPCR Master Mix (Bioneer). Information on primers and binding sites following the described supplemental data are provided in Figs. S2 and S3.

### Cytokine profiling using flow cytometric analysis

MDA-MB-231-derived CSCs were seeded in an ultra-low attachment 6-well plate. After 6 days, the cells were treated with 1 µM (+)-JQ1, 0.1 µM ARV825, 2 µM Verteporfin, 50 µM calcitriol, and 20 µM caffeic acid for 24 h. The cytokine profiling of cells was assessed in a supernatant culture medium using Human Inflammatory Cytokine Cytometric Bead Array (CBA) (BD Biosciences) and FACS. The procedures followed the manufacturer’s protocol. The samples were measured by flow cytometry (Accuri C6, BD Biosciences). CBA data were analyzed and quantitated using BD FCAP array software.

### Cytokine quantitation using enzyme-linked immunosorbent assay (ELISA)

MC38 cells (2.5 × 10^5^ cells/mL) were cultured in a 6-well plate. MC38 cells were treated with 200 µM THF for 18 h. IL-6 was measured in a supernatant culture medium. The amount of IL-6 was measured by ELISA MAX™ Deluxe Set Mouse IL-6 (BioLegend, San Diego, CA, USA) according to the manufacturer’s protocol. The absorbance was measured using a VersaMax ELISA microplate reader (Molecular Devices).

### Mice

Female C57BL/6 and BALB/c mice, 5 or 6 weeks old (Samtako, Osan-si, Gyeonggi-do, South Korea) were used for animal studies. The animal studies were approved the by Institutional Animal Care and Use Committee (IACUC-2022-031) of Jeju National University.

### Tumor subcutaneous induction and treatment

MC38 and 4T1 cells were harvested at four passages. After being washed, the cells were resuspended in the Matrigel matrix media (Corning Inc., NY, USA). Mice were shaved at the right flank before cancer cell injection. MC38 cells (5 × 10^5^ cells/mice) and 4T1 cells (5 × 10^5^ cells/mice) were injected subcutaneously into the right hind flank of each mouse. From day 6 after tumor inoculation, tumor-bearing mice were injected with THF (10 mg/kg, DMSO) every 2–3 days. The mice were randomly divided into different experimental groups. Tumor length and width were measured every 2–3 days, and the tumor volume was calculated using the formula: (length × width^2^)/2.

### Subcutaneous tumor and TDLN resection and sample preparation

To identify the TDLNs of mice, 100 µL of 1% Evans blue (Sigma-Aldrich, St. Louis, MO, USA) with saline was injected into the subcutaneous tumor. TDLNs of the right inguinal were visually identified after treatment with Evans blue for 60 min. When subcutaneous tumors reached a volume of approximately 4000 m^3^, mice were sacrificed. Subcutaneous tumors and dyed TDLNs were resected, and cell suspensions were prepared mechanically. After being harvested, single-cell suspensions were prepared for use in FACS analysis. Small cut tumor pieces and TDLNs were incubated with ACCUMAX (Innovative Cell Technologies, San Diego, CA, USA) at room temperature for 1 h with shaking. Then, the tissues were meshed and filtered through a 70-µm cell strainer. The cell suspension was harvested, and a single-cell suspension was obtained.

## Results

### BRD4 is upregulated in breast CSCs and regulates breast CSC formation

**Fig. 1:**
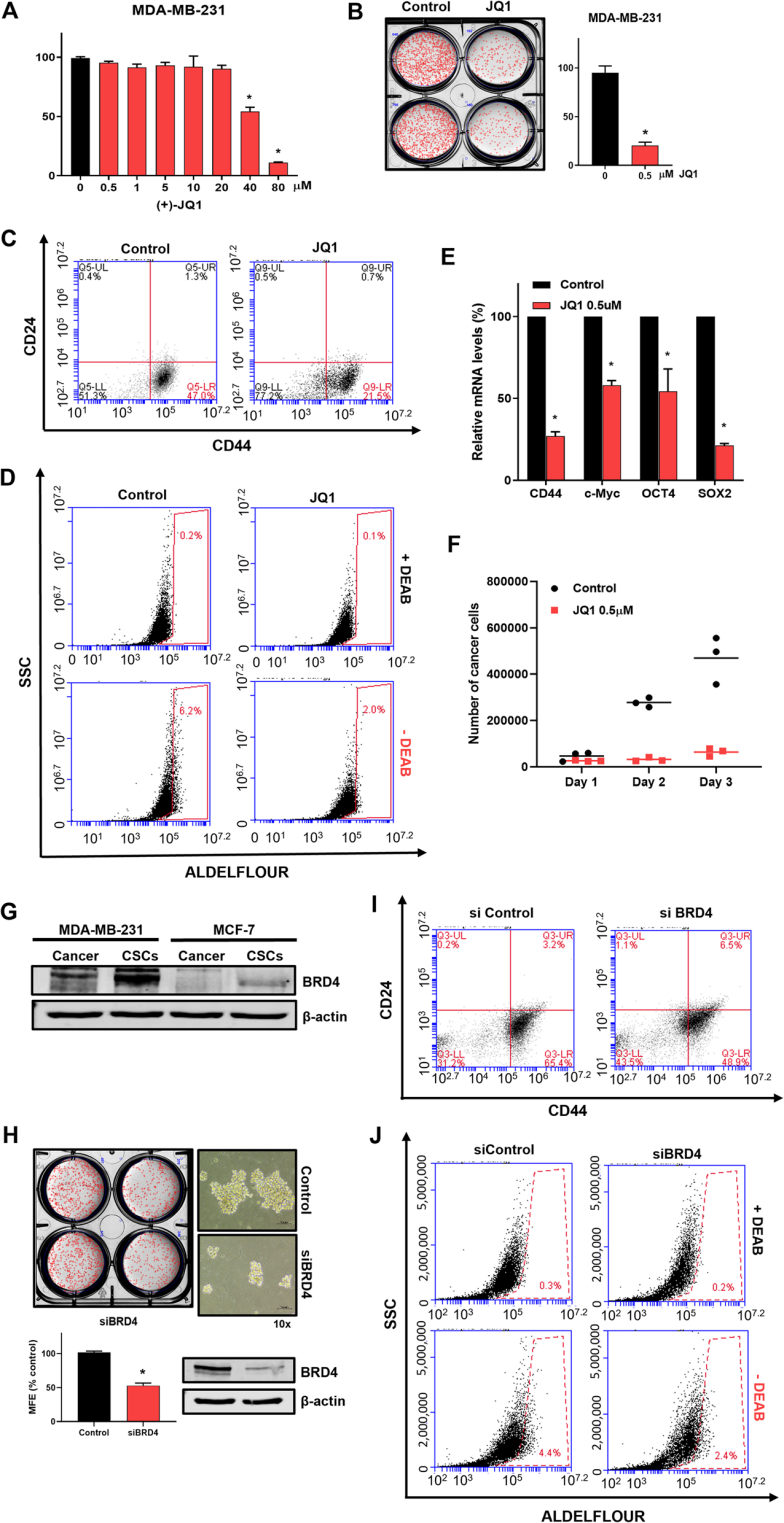
BRD4 inhibitor, (+)-JQ1, reduces triple-negative breast cancer growth and mammosphere formation. **A** Effect of JQ1 on the proliferation of MDA-MB-231 cells. The cells were cultured with the indicated concentration range of JQ1 for 24 h. Cell viability was measured using the MTS assay. **B** Inhibitory effect of mammosphere formation by JQ1. Treatment with 0.5 µM JQI reduced mammosphere formation to 20%. **C** Breast CSC marker, CD44^+^/CD24^−^ expression in MDA-MB-231 cells. The cells were treated with 1 µM JQ1 for 1 day. CD44^+^/CD24^−^ expression was evaluated using a flow cytometer. **D** CSC marker, ALDH expression in MDA-MB-231 cells. The cells were treated with 1 µM JQ1 for 1 day. ALDH expression was measured using the ALDEFLUOR assay kit and a flow cytometer, as described in the “Materials and methods” section. **E** CSC marker gene expressions in MDA-MB-231 cells treated with JQ1. The cells were treated with 0.5 µM JQ1 for 18 h. The mRNA levels of CD44, c-Myc, OCT4, and SOX2 were measured by reverse-transcription quantitative polymerase chain reaction. **F** Inhibitory effect of JQ-1 on mammosphere growth. JQ1-treated mammospheres were divided into single cells, and equal numbers of cells were cultured. The number of cells was analyzed daily for 3 days by counting. **G** Difference in BRD4 protein expression in breast cancer cells and mammospheres. BRD4 protein expression was analyzed in cancer cells and mammospheres derived from MDA-MB-231 and MCF-7 cells by Western blot, as described in the “Materials and methods” section. **H** Effect of BRD4 on mammosphere formation. After BRD4 knockdown using siRNA, mammosphere formation was reduced, as shown in the photos and graphs. The knockdown of BRD4 was verified by Western blot, and images of the mammosphere (right) were taken at ×10 magnification. **I** CSC marker, CD44^+^/CD24^−^ expression on BRD4-knockdown cells. The cells were treated with siBRD4 for 2 days. CD44^+^/CD24 expression was evaluated using a flow cytometer. **J** CSC marker, ALDH, expression on BRD4-knockdown cells. The cells were treated with siBRD4 for 2 days. ALDH expression was measured using the ALDEFLUOR assay kit and a flow cytometer. Experiment values are represented as the mean ± SD of triplicates. Compared with the control as determined by student’s t-test or one-way ANOVA with Dunnett’s multiple comparisons tests, * *p* < 0.05

Initially, to determine the function of BRD4 in BCSCs, BRD4 inhibitor, JQ1, was explored at breast CSC formation with the main focus on BC stemness. MDA-MB-231 cancer cells were treated with JQ1 for 24 h. JQ1 inhibited the proliferation of MDA-MB-231 cancer cells at 40 µM (Fig. 1A). To examine a CSC-suppressing effect of the JQ1, a mammosphere formation assay was performed. JQ1 decreased the sphere size and the number of tumorspheres derived from MDA-MB-231 at 0.5 µM (Fig. 1B). The results indicated that JQ1 suppresses mammosphere formation. The CD44^+^/CD24^−^ and ALDH-expressing populations represent breast CSC populations and use as breast CSC markers. MDA-MB-231 cancer cells were cultured with or without JQ1 for 24 h, and the CD44^+^/CD24^−^ and ALDH-expressing subpopulations were determined. JQ1 decreased the CD44^+^/CD24^−^ expressing subpopulation from 47 to 21.5% (Fig. 1C). JQ1-treated cells had reduced ALDH expression from 6.2 to 2% (Fig. 1D).

To examine the expression levels of CSC-specific genes by JQ1 and the inhibitory effect of JQ1 on mammosphere proliferation through BRD4 inhibition, MDA-MB-231 cell and mammospheres were treated with JQ1. JQ1 inhibits *Oct4*, *CD44*, *Sox2*, and *c-myc* genes (Fig. 1E) and inhibited mammosphere proliferation (Fig. 1F). OCT4 and SOX2 protein expressions were reduced on JQ1 treatment (Fig. S4). To examine the effects of BRD4 on CSC formation, we checked the BRD4 levels of BCs and mammospheres. BRD4 is upregulated in breast CSCs derived from MCF7 and MDA-MB-231 cells (Fig. 1G). siRNA silencing of BRD4 significantly reduced the mammosphere formation of MDA-MB-231 cells (Fig. 1H). The silencing of BRD4 of MDA-MB-231 cells with siRNA of BRD4 decreased the CD44^+^/CD24^−^-expressing subpopulation from 65.4 to 48.9% (Fig. 1I) and ALDH-expressing population from 4.4 to 2.4% (Fig. 1J).

**Fig. 2:**
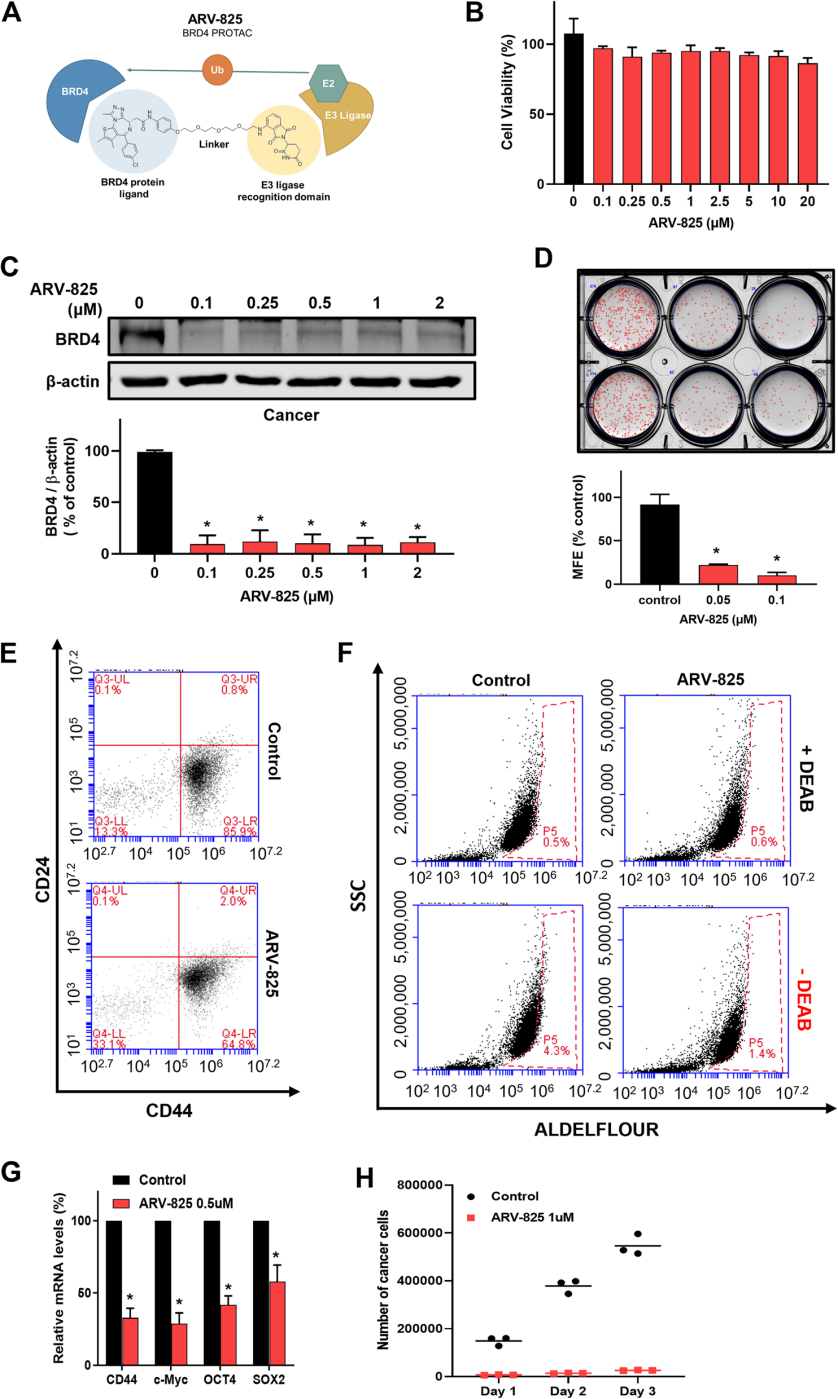
BRD4 degrader ARV-825 inhibited mammosphere formation. **A** Molecular structure of BRD4 degrader ARV-825. **B** Proliferation assay using ARV-825 on MDA-MB-231 cells. The cells were cultured with increasing concentration range of ARV-825 for 24 h. **C** Effect of ARV-825 on BRD4 degradation. The cells were cultured using the indicated concentrations of ARV-825 for 24 h. **D** Effect of BRD4 degrader ARV-825 on mammosphere formation. ARV-825-treated mammosphere formation was reduced, as shown in the photos and graphs. Experiment values are represented as the mean ± SD of triplicates. **E** CSC marker, CD44^+^/CD24^−^ expression on ARV-825-treated cells. The cells were treated with ARV825 for 24 h. CD44^+^/CD24 expression was evaluated using a flow cytometer. **F** CSC marker, ALDH expression on ARV-825-treated cells. The cells were treated with ARV825 for 24 h. ALDH expression was measured using the ALDEFLUOR assay kit and a flow cytometer. **G** CSC-related gene expression on ARV-825-treated cells. The cells were treated with 0.5 µM ARV-825 for 18 h. The mRNA levels of CD44, c-Myc, OCT4, and SOX2 were measured by reverse-transcription quantitative polymerase chain reaction. **H** Inhibitory effect of ARV-825 on mammosphere growth. ARV825-treated mammospheres were divided into single cells, and equal numbers of cells were cultured. The number of cells was analyzed daily for 3 days by counting. Experiment values are represented as the mean ± SD of triplicates. Compared with the control as determined by student’s t-test or one-way ANOVA with Dunnett’s multiple comparisons tests, * *p* < 0.05

ARV-825 is a PROTAC BRD4 inhibitor that recruits BRD4 to the E3 ubiquitin ligase, leading to fast, efficient, and prolonged degradation of BRD4 [27]. We checked BRD4 function on breast CSC formation using ARV-825 and BRD4 proteolysis targeting chimera (PROTAC) degrader (Fig. 2A). ARV-825 degraded BRD4 protein and inhibited CSC formation at 0.1 µM concentration without cell death of BCs (Fig. 2B–D). The degradation of BRD4 of MDA-MB-231 cells with AVR-825 reduced the CD44^+^/CD24^−^-expressing subpopulation from 85.9 to 64.8% (Fig. 2E) and ALDH-expressing population from 4.3 to 1.4% (Fig. 2F). CSC-specific gene expression and mammosphere proliferation were assayed using ARV-825. The result represents that ARV-825 inhibits *Oct4*, *CD44*, *Sox2*, and *c-myc* (Fig. 2G) and inhibited mammosphere proliferation (Fig. 2H).

### BRD4 regulates the gene expression of PD-L1 and PD-L1 modulates breast CSC formation

**Fig. 3:**
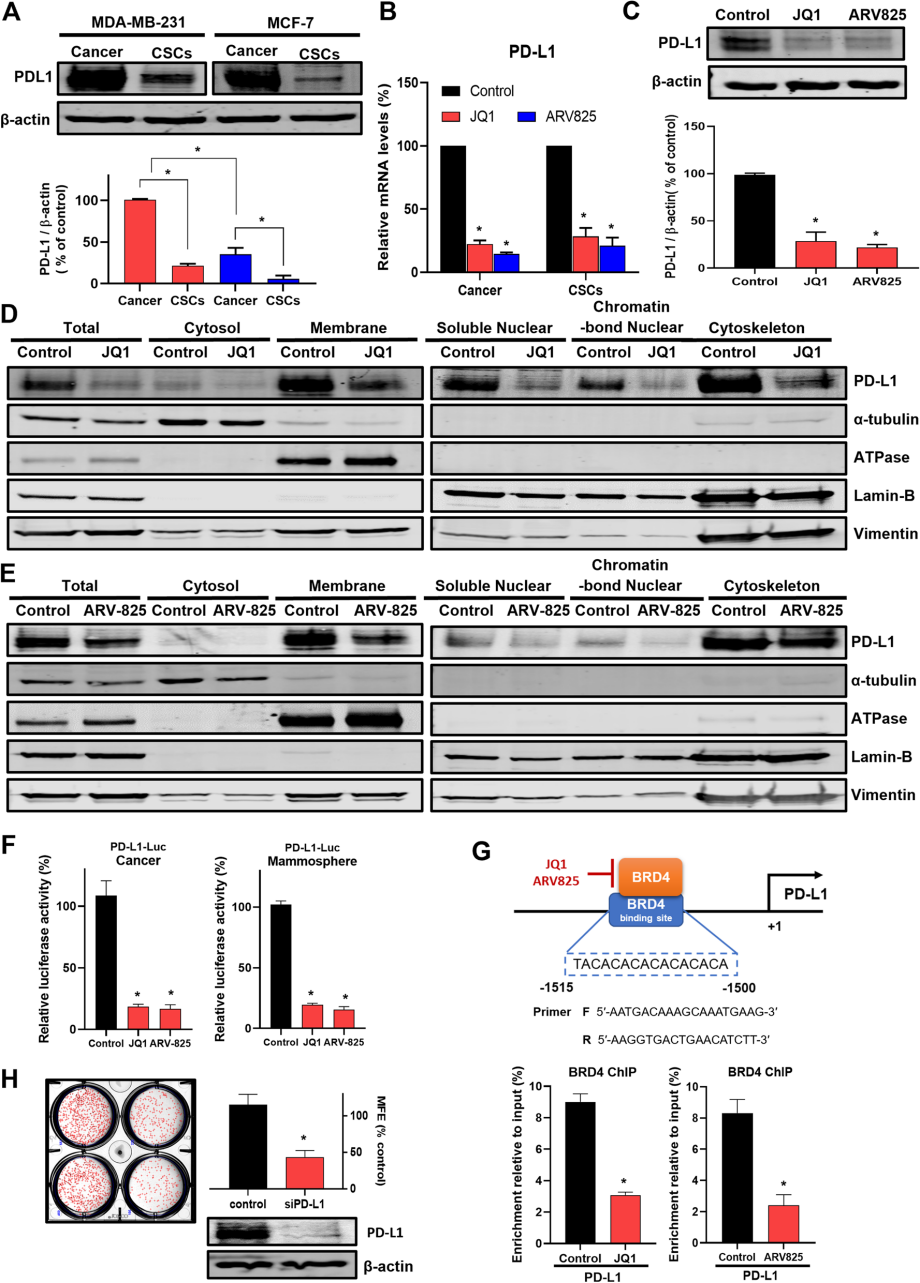
BRD4 degrader ARV-825 inhibited PD-L1 expressions, and PD-L1 regulated mammosphere formation. **A** PD-L1 protein expression in breast cancer cells and mammospheres. PD-L1 protein expressions were analyzed in cancer cells and mammospheres derived from MDA-MB-231 and MCF-7 cells by Western blot, as described in the “Materials and methods” section. **B** Transcriptional regulation of PD-L1 genes by BRD4 inhibitor JQ1 and ARV-825-treated MDA-MB-231 cells and mammospheres. The cells were treated with 0.5 µM JQ1 and 0.5 µM ARV-825 for 18 h. The mRNA level of PD-L1 was measured using reverse-transcription quantitative polymerase chain reaction. **C** PD-L1 protein levels in mammospheres after treatment of the BRD4 inhibitor and ARV-825. The mammospheres were treated with 1 µM JQ1 and 0.5 µM ARV-825 for 24 h. **D**, **E** Fractional analysis of PD-L1 protein expression in mammospheres. Cell lysate was fractionated using an isolation kit, as described in the “Materials and Methods” section. The mammospheres of MDA-MB-231 cells were treated with 1 µM JQ1 or 0.5 µM ARV-825 for 24 h. The fractions were analyzed by Western blot with PD-L1 antibody, and subcellular location markers were detected with antibodies (α-tubulin, ATPase, Lamin-B, and vimentin). **F** PD-L1 reporter luciferase assay using MDA-MB-231 cells and mammospheres. PD-L1 reporter plasmid was transfected into cancer and mammosphere, and cells were then treated with 1 µM JQ1 and 0.5 µM ARV-825 for 24 h. The cells were lysed, and luciferase was assayed as described in the “Materials and methods” section. **G** Chromatin immunoprecipitation (ChIP) assay on the promoter of PD-L1 gene using anti-BRD4. The binding site of BRD4 on the CD274 (PD-L1) gene is shown in Fig. 3G. Mammospheres were treated with 1 µM JQ1 or 0.5 µM ARV825. ChIP analysis using an antibody against BRD4 and the negative control IgG. **H** Effect of PD-L1 on mammosphere formation. Cultured MDA-MB-231 cells were treated with siPD-L1 for 2 days. The mammospheres derived from siPD-L1 cells were cultured for 7 days. PD-L1 knockdown was verified by Western blot. Experiment values are represented as the mean ± SD of triplicates. Compared with the control as determined by student’s t-test or one-way ANOVA with Dunnett’s multiple comparisons tests, * *p* < 0.05

BET inhibitor, JQ-1, has shown suppression of PD-L1 expression levels in ovarian cancer and represents a treatment strategy for targeting PD-L1 expression [28]. We checked PD-L1 and the effect of the BRD4 inhibitor (JQ1) and degrader (ARV825). The expressions of PD-L1 of BC cells and CSCs were analyzed, and they expressed PD-L1 (Fig. 3A). JQ1 and ARV825 decreased the transcript and protein levels of PD-L1 (Fig. 3B, C). A previous paper showed that PD-L1 is located in the cellular membrane and nucleus through endocytosis and nucleocytoplasmic transport pathways [29]. A research group reported that nuclear PD-L1 is independent of immune checkpoint function and inhibited cell proliferation, colony formation, and tumor growth through sister chromatid cohesion of cancer cells [30]. In addition, we confirmed that the morphology of the chromosome was changed when treated with verteporfin and siRNA of PD-L1 (Fig. S1). Our data show that JQ1 and ARV-825 reduced the total, membrane, cytoskeleton, soluble nuclear, and chromatin-bond nuclear levels of PD-L1 protein (Fig. 3D, E). Initially, we checked the transcript levels of PD-L1 under BRD4 inhibitor and degrader treatment. The PD-L1-Luc reporter assay showed that JQ1 and ARV825 inhibit PD-L1 promoter activity based on the luciferase reporter assay (Fig. 3F) and suppressed the BRD4 binding of PD-L1 promoter by BRD4-ChIP assay (Figs. 3G and S2). BRD4 inhibition and degradation inhibit promoter activity and BRD4 binding of PD-L1 promoter. To examine the effects of PD-L1 on CSC formation, we checked CSC formation through siRNA-mediated silencing PD-L1. The siRNA of PD-L1 inhibits the formation of breast CSCs derived from MDA-MB-231 cancer cells (Fig. 3H). We found that nuclear PD-L1 regulates breast CSC formation.

### Nuclear PD-L1 modulated breast CSC formation through the regulation of the *RelB* gene

**Fig. 4:**
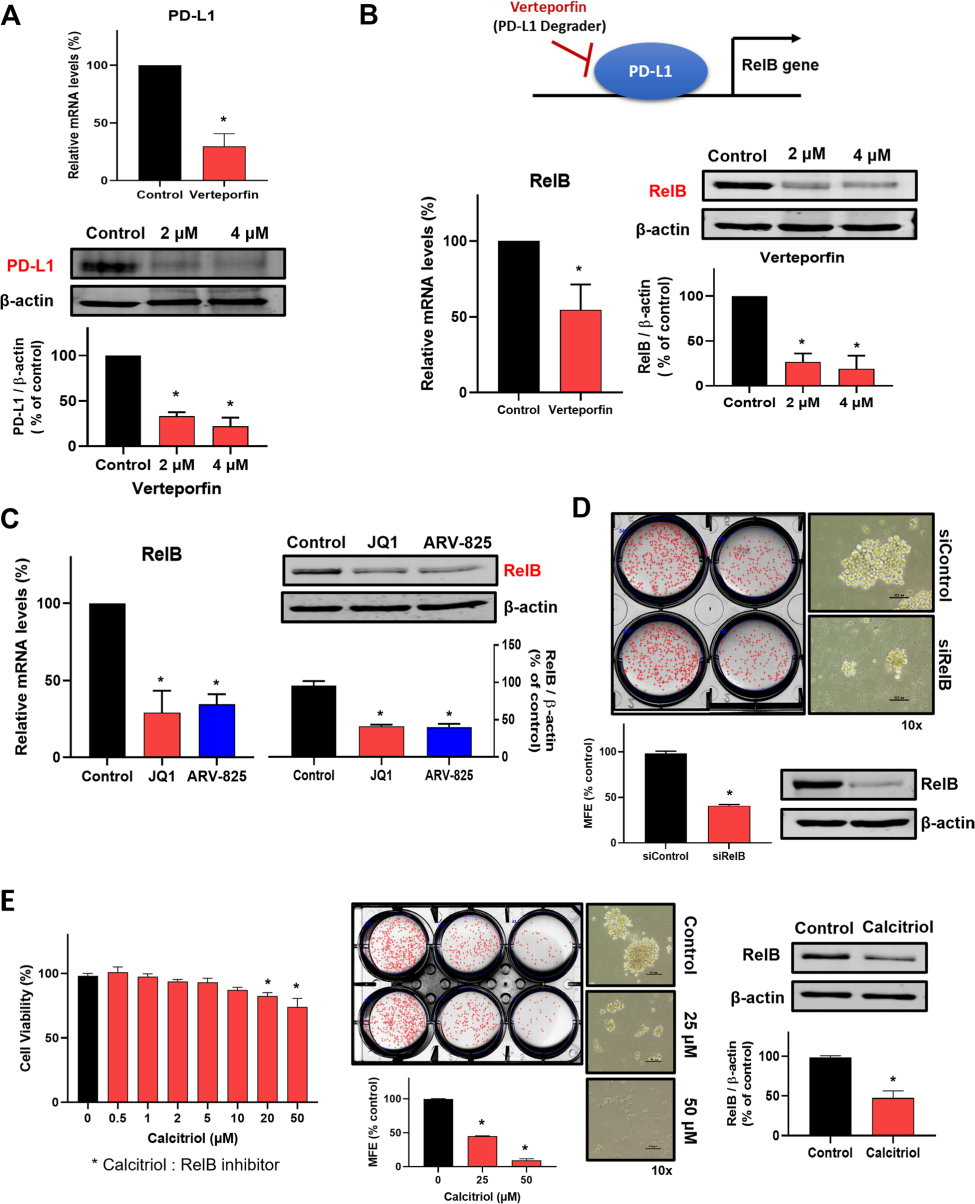
PD-L1-regulated *RelB* gene-regulated mammosphere formation. **A** Regulation of PD-L1 expression in mammosphere by verteporfin, a PD-L1 degrader. The cells were treated with 2 µM verteporfin for 18 h. The mRNA levels of PD-L1 were measured by reverse-transcription quantitative polymerase chain reaction (RT-qPCR). The protein expression of PD-L1 was detected by Western blot. The cells were treated with 2 and 4 µM verteporfin for 24 h. **B** Transcriptional regulation of RelB by PD-L1 inhibition. After treatment of 2 µM verteporfin for 18 h, the mRNA levels of RelB were measured by RT-qPCR. The cells were treated with 2 and 4 µM verteporfin for 24 h, and the protein expression of RelB was detected by Western blot. **C** Transcriptional and translational regulations of RelB through BRD4 inhibition. The mRNA levels of RelB were measured by RT-qPCR. The cells were treated with 1 µM JQ1 or 0.5 µM ARV-825 for 18 h. The protein expression of RelB was detected by Western blot. The cells were treated with 1 µM JQ1 or 0.5 µM ARV-825 for 24 h. **D** Regulation of mammosphere formation by RelB. Cultured MDA-MB-231 cells were transfected with si-RelB for 2 days. The mammospheres derived from si-RelB-transfected cells were incubated for 7 days. RelB knockdown was verified by Western blot. The mammosphere images (right) were taken at ×10 magnification. **E** Inhibitory effect of RelB inhibitor calcitriol on mammosphere formation. Proliferation of calcitriol-treated cells was measured using the MTS assay. The cells were treated with an increasing concentration range of calcitriol for 24 h. Mammospheres derived from MDA-MB-231 cells were treated with the indicated concentrations of calcitriol for 7 days. The RelB-inhibitory effect of calcitriol was verified by Western blot. Mammosphere images (right) were taken at ×10 magnification. Experiment values are represented as the mean ± SD of triplicates. Compared with the control as determined by student’s t-test or one-way ANOVA with Dunnett’s multiple comparisons tests, * *p* < 0.05

Nuclear PD-L1 regulated gene expression of NF-κB signaling (BIRC3, RelB, and TRAP1), major histocompatibility complex (MHC) class I (human leukocyte antigen (HLA)-A, HLA-B, and HLA-H), and immune checkpoint (PD-L2, VISTA, and B&-H3) [29]. The NF-κB pathway is a crucial factor in CSC formation and therapeutic target [26, 31]. As verteporfin decreases the intrinsic and interferon-induced PD-L1 expressions of six cancer cell lines [32], we checked the transcript and protein levels of PD-L1 under verteporfin treatment. Verteporfin reduced the transcript and protein levels of PD-L1 at 2 µM Fig. 4A). As nuclear PD-L1 regulated gene expression of RelB and the NF-κB pathway is a crucial factor of CSC formation, we checked the transcript and protein levels of RelB under verteporfin treatment. Verteporfin reduced the transcript and protein levels of RelB at the same concentration that reduced PD-L1 (Fig. 4B). To investigate the function of RelB in breast CSC formation, we used BRD4 inhibitor (JQ1) and degrader (RV825). JQ1 and ARV825 reduced the transcript and protein levels of RelB (Fig. 4C). We investigated RelB function on CSC formation through RelB silencing. The siRNA-mediated silencing of RelB inhibits the formation of breast CSCs derived from MDA-MB-231 cells (Fig. 4D). We used RelB inhibitor, 1α, 25-dihydroxy vitamin D3, also known as calcitriol [33]. Calcitriol reduced the protein levels of RelB and inhibits cell proliferation at 20 µM and mammosphere formation at 25 µM (Fig. 4E). Our data show that RelB regulates breast CSC formation.

### RelB binds with p65 and RelB/p65 complex regulated transcripts and secretory IL6

**Fig. 5:**
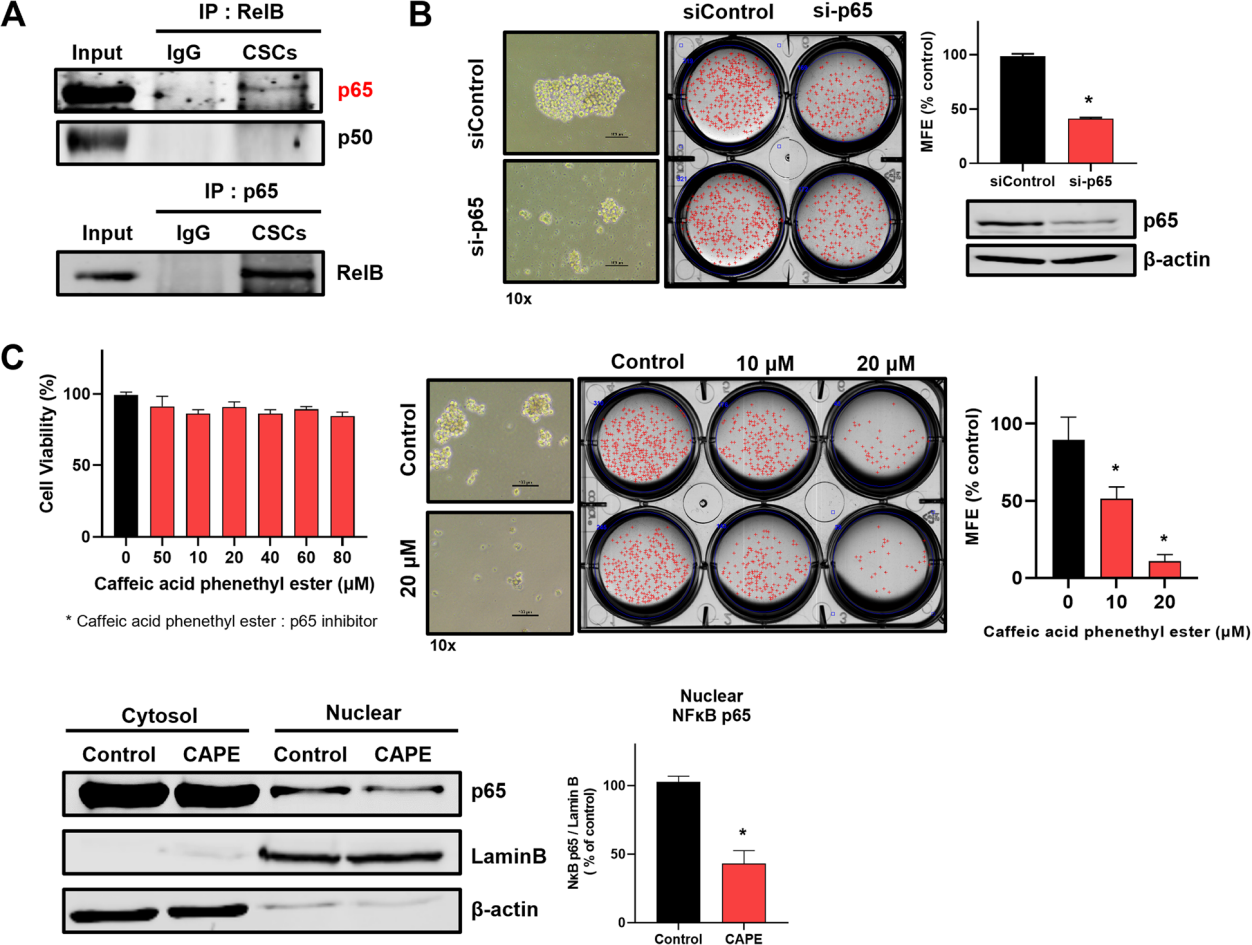
RelB/p65 complex regulated mammosphere formation. **A** CoIP assay. CoIP assay was performed in mammospheres derived from MDA-MB-231 cells. The cell lysate was incubated with the RelB antibody or corresponding IgG. The immunoprecipitants were blotted with p65 or p50. The RelB/p65 complex was verified through the IP assay of p65. **B** Mammosphere formation regulation of p65. Cultured MDA-MB-231 cells were transfected with si-p65 for 2 days. Mammospheres derived from si-p65-transfected cells were incubated for 7 days. p65 knockdown was verified by Western blot, and mammosphere images (right) were taken at ×10 magnification. **C** Inhibitory effect of p65 inhibitor caffeic acid phenethyl ester (CAPE) in triple-negative breast cancer cells and mammospheres. The proliferation of CAPE-treated cells was measured using the MTS assay. The cells were treated with increasing concentration range of CAPE for 24 h. Mammospheres derived from MDA-MB-231 cells were given indicated concentrations of CAPE for 7 days. The RelB-inhibitory effect of CAPE was verified by Western blot of nuclear fraction. 20 µM CAPE was used to perform Western blot. Experiment values are represented as the mean ± SD of triplicates. Compared with the control as determined by student’s t-test or one-way ANOVA with Dunnett’s multiple comparisons tests, * *p* < 0.05

**Fig. 6:**
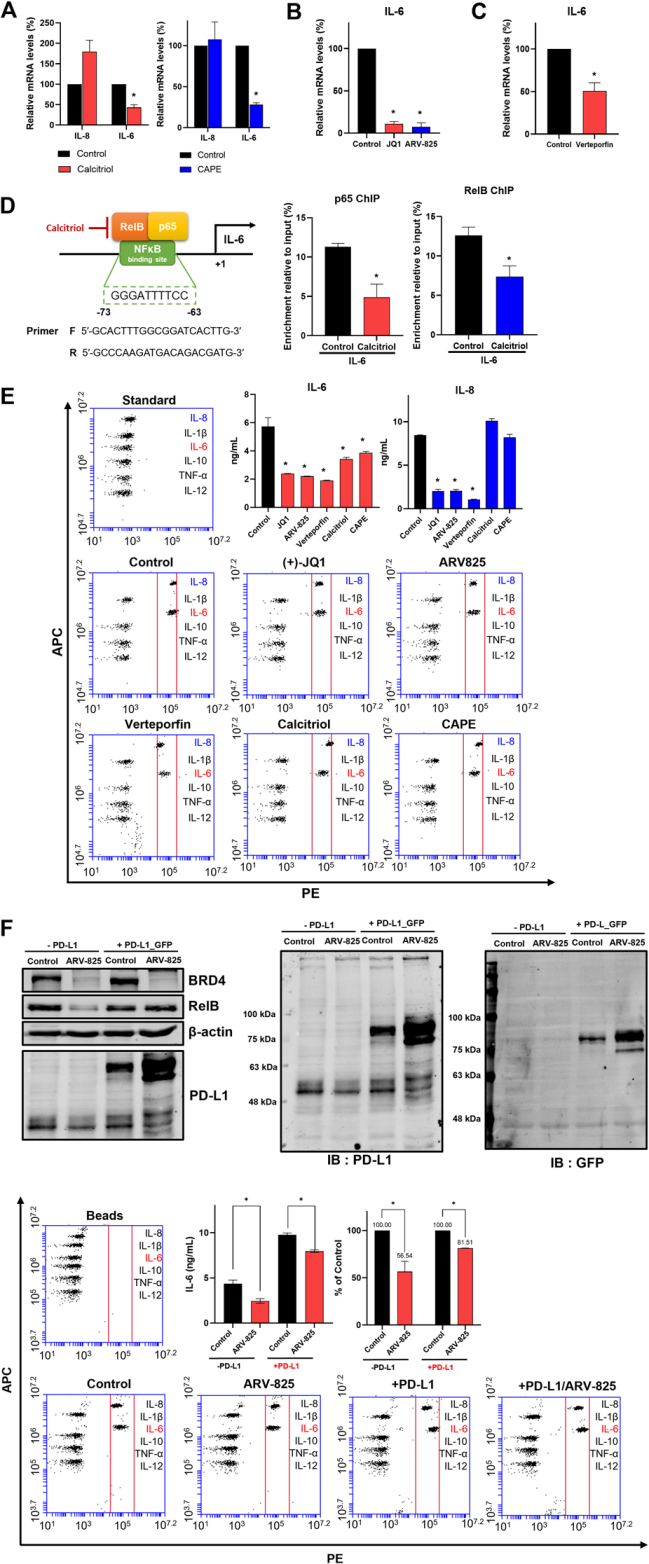
RelB/p65 complex regulated IL-6 transcriptional expression. **A** Effect of calcitriol and caffeic acid phenethyl ester on interleukin (IL)-6 and IL-8 of mammospheres. Mammospheres were treated with 40 µM calcitriol or 20 µM caffeic acid phenethyl ester for 18 h. mRNA levels of IL-6 and IL-8 were analyzed by reverse-transcription quantitative polymerase chain reaction (RT-qPCR). **B** Effect of BRD4 inhibitor on IL-6 gene expression on mammosphere. Mammospheres were treated with 1 µM JQ1 or 0.5 µM ARV-825 for 18 h. mRNA levels of IL-6 were analyzed by RT-qPCR. **C** Effect of PD-L1 inhibitor on IL-6 gene expression on mammospheres. Mammospheres were treated with 4 µM verteporfin for 18 h. mRNA levels of IL-6 were analyzed by RT-qPCR. **D** ChIP assay on the promoter of IL-6. The binding site of NF-kB on IL-6 promoter is shown in Fig. 6D. Mammospheres were treated with 40 µM calcitriol. ChIP analysis used an antibody against p65 or RelB. The negative control used was IgG. **E** Cytokine profiling in mammospheres. Cytokine profiling was performed at the drug-treated mammosphere culture medium, and the drug concentrations were as follows: 1 µM JQ1, 0.5 µM ARV-825, 4 µM verteporfin, 40 µM calcitriol, or 20 µM caffeic acid phenethyl ester. The amounts of IL-6 and IL-8 were quantified using a flow cytometer. **F** Rescue experiment using PD-L1 overexpression plasmid to confirm the existence of the BRD4/PD-L1/RelB/IL-6 axis. Protein expression regulation by ARV-825 in breast cancer. The PD-L1 expression plasmid vector, pEGFP-N1/PD-L1 was transfected into MDA-MB-231 cells and the transfected cancer cells were treated with 0.5 µM ARV-825 for 24 h. The protein expressions of BRD4, PD-L1, and RelB were detected by Western blot. Cytokine profiling was performed at ARV-825-treated cancer cell culture medium. The amount of IL-6 was quantified using a flow cytometer. Experiment values are represented as the mean ± SD of triplicates. Experiment values are represented as the mean ± SD of triplicates. Compared with the control as determined by student’s t-test or one-way ANOVA with Dunnett’s multiple comparisons tests, * *p* < 0.05

RelB/p65 (RelA) complex promotes NF-κB target genes such as TNF and IL-6 in ER-negative BC [34]. We examined the interaction of RelB and p65 to understand the CSC regulation of RelB. Figure 5 A shows that RelB and p65 (RelA) interact with each other. The downregulation of p65 using siRNA of p65 reduced the mammosphere formation of BC (Fig. 5B). We used caffeic acid phenethyl ester (CAPE) to investigate CSC regulation by nuclear p65 activity. CAPE has strong inhibitory effects on NF-κB activation through the inhibition of NF-κB p65 phosphorylation [35]. We assayed cell proliferation, mammosphere formation, and nuclear localization of p65 using CAPE (Fig. 5C). Our results showed that CAPE did not change cell proliferation, but inhibited mammosphere formation and nuclear localization of p65. CSC formation is regulated by the nuclear location of p65, the NF-κB component. Cytokines, including IL-6 and IL-8, regulated by NF-κB proteins regulated breast CSC population [36]. The levels of CSC-regulating cytokines, IL-6 and IL-8, were examined under a BRD4 inhibitor, PD-L1 inhibitor, RelB inhibitor, and p65 inhibitor treatments. The RelB inhibitor (calcitriol) and p65 inhibitor (CAPE) only inhibited the transcript of IL-6 (Fig. 6A). The BRD4 inhibitor (JQ1) and PD-L1 inhibitor (verteporfin) also inhibited the transcript of IL-6 (Fig. 6B, C). Then, we performed the ChIP assay to identify RelB and p65 binding of the IL-6 promoter using a PCR primer set spanning the IL-6 promoter (Figs. 6D and S3). RelB inhibitor (calcitriol) endogenously reduced the IL-6 promoter binding affinity of RelB and p65. The *IL-6* gene of breast CSCs is regulated by RelB and p65 protein through the binding of the IL-6 promoter. Secretory IL-6 and IL-8 play essential roles in mammosphere formation. To assess the production of secretory IL-6 and IL-8, we performed a human inflammatory cytokine assay on mammosphere-cultured broth using cytokine assay beads. The human inflammatory cytokine data indicated that inhibitors only reduce the production of secretory IL-6, not IL-8 (Fig. 6E). We performed the rescue experiments using the PD-L1 overexpression plasmid to understand the role of the existence of the BRD4/PD-L1/RelB/IL-6 axis in breast CSCs. For the rescue of BRD4-dependent PD-L1 degradation, the PD-L1_GFP expression vector was transfection into breast cancer cells with/without ARV-825 (BRD4 degrader). BRD4 degrader reduced BRD4, PD-L1, RelB, and IL-6 levels and overexpression of PD-L1 of ARV-825 treated breast cancers increased reduced PD-L1, RelB, and IL-6 levels (Fig. 6F). Our experiments showed the existence of the BRD4/PD-L1/RelB/IL-6 in breast CSCs.

### Natural product, THF, and BRD4 inhibitor inhibit CSC formation through the downregulation of the BRD4/PD-L1/RelB/IL-6 axis

**Fig. 7:**
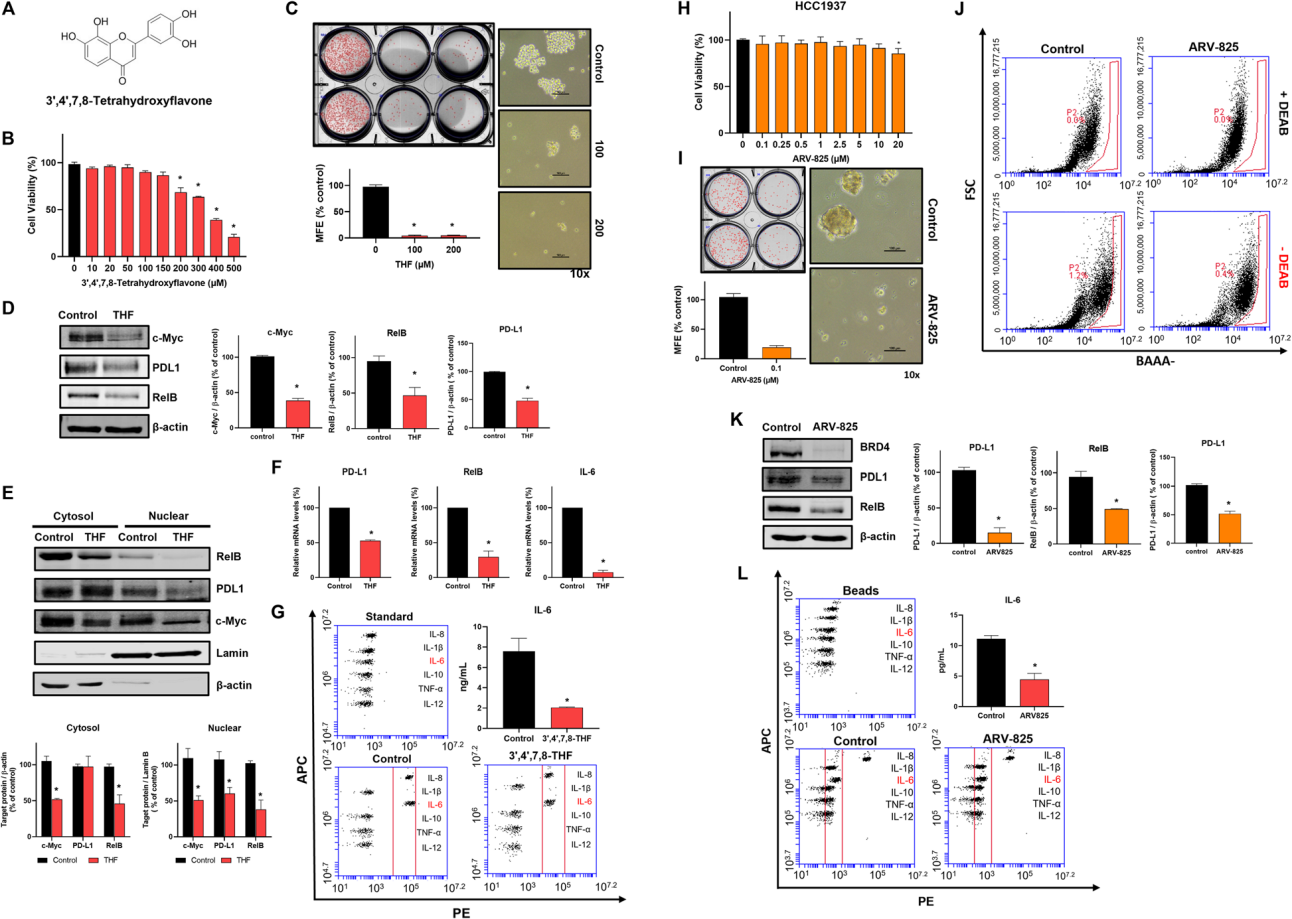
BRD4 inhibitory natural compound 3',4',7,8-tetrahydroxyflavone (THF) regulated mammosphere formation derived from MDA-MB-231 and ARV-825 inhibits mammosphere formation derived from HCC1937 through the inhibition of BRD4/PD-L1/RelB. **A** Structure of THF. **B** Inhibitory effect of THF on the proliferation of MDA-MB-231 cells. The cells were cultured with the indicated concentration range of THF for 24 h. Cell viability was measured using the MTS assay. **C** Inhibitory effect of THF on mammosphere. The mammospheres derived from MDA-MB-231 cells were treated with the indicated concentrations of THF for 7 days. Mammosphere images (right) were taken at ×10 magnification. **D** Protein expression regulation by THF in mammospheres. The protein expressions of c-Myc, PD-L1, and RelB were detected by Western blot. The cells were treated with 100 µM THF for 24 h. **E** Protein expression regulation of THF in nuclear and cytosolic fractions of mammospheres. The protein expressions of c-Myc, PD-L1, and RelB were detected by Western blot. The cells were treated with 100 µM THF for 24 h. **F** Transcriptional regulation of THF on mammosphere. The cells were treated with 100 µM THF for 18 h. mRNA levels of PD-L1, RelB, and IL-6 were analyzed using reverse-transcription quantitative polymerase chain reaction. **G** Cytokine profiling in mammospheres. Cytokine profiling was performed at THF (100 µM)-treated mammosphere culture medium. The amount of IL-6 was quantified using a flow cytometer. **H** Proliferation assay using ARV-825 on breast cancer cell line, HCC1937 cells. The cells were cultured with an increasing concentration range of ARV-825 for 24 h. **I** Effect of BRD4 degrader ARV-825 on mammosphere formation. ARV-825-treated cells reduced mammosphere formation, as shown in the photos and graphs. Experiment values are represented as the mean ± SD of triplicates. **J** CSCs marker, ALDH expression on ARV-825-treated cells. The cells were treated with ARV-825 for 24 h. ALDH expression was measured using the ALDEFLUOR assay kit and a flow cytometer. **K** Protein expression regulation of ARV-825 in mammosphere derived from HCC1937. The protein expressions of c-Myc, PD-L1, and RelB were detected by Western blot. The cells were treated with 0.1 µM ARV-825 for 24 h. **L** IL-6 level of mammospheres under ARV-825 treatment. The level of IL-6 was examined in the ARV-825 (0.1 µM)-treated mammosphere culture medium. The amount of IL-6 was quantified using a flow cytometer. Experiment values are represented as the mean ± SD of triplicates. Compared with the control as determined by student’s t-test or one-way ANOVA with Dunnett’s multiple comparisons tests, * *p* < 0.05

THF is a natural product found in *Acacia confuse*. THF was known as a novel and potent selective BRD4 inhibitor [37]. To find a natural compound for targeting breast CSCs, we selected THF, a BRD4 inhibitor (Fig. 7A). THF inhibits cell proliferation at 200 µM and mammosphere formation at 100 µM (Fig. 7B, C). THF downregulates not only c-Myc, PD-L1, and RelB in whole cells (Fig. 7D) but also the nuclear protein levels of c-Myc, PD-L1, and RelB (Fig. 7E). THF reduced the transcripts (Fig. 7F) and secretory IL-6 (Fig. 7G). We have examined the effect of a selective BRD4 inhibitor for targeting breast CSCs using the TNBC line HCC1937. ARV-825, BRD4 degrader inhibited CSCs formation at 0.1 µM concentration without cell death of BCs (Fig. 7H, I). HCC1937 cells with ARV-825 reduced the ALDH-expressing population from 1.2 to 0.4% (Fig. 7J). To confirm the biochemical function of ARV-825 on HCC1937 cells, we analyzed the levels of BRD4, PD-L1, RelB, and IL-6. ARV-825 reduced total protein levels of BRD4, PD-L1, RelB, and IL-6 in mammospheres derived from HCC1937 cells (Fig. 7K, L). Our data showed that BRD4/PD-L1/RelB/IL-6 axis regulates breast CSC formation and our suggestion was confirmed by THF and ARV-825.

### Functional assay of THF as an anti-CSC agent using MC-38 murine colon carcinoma cells

**Fig. 8:**
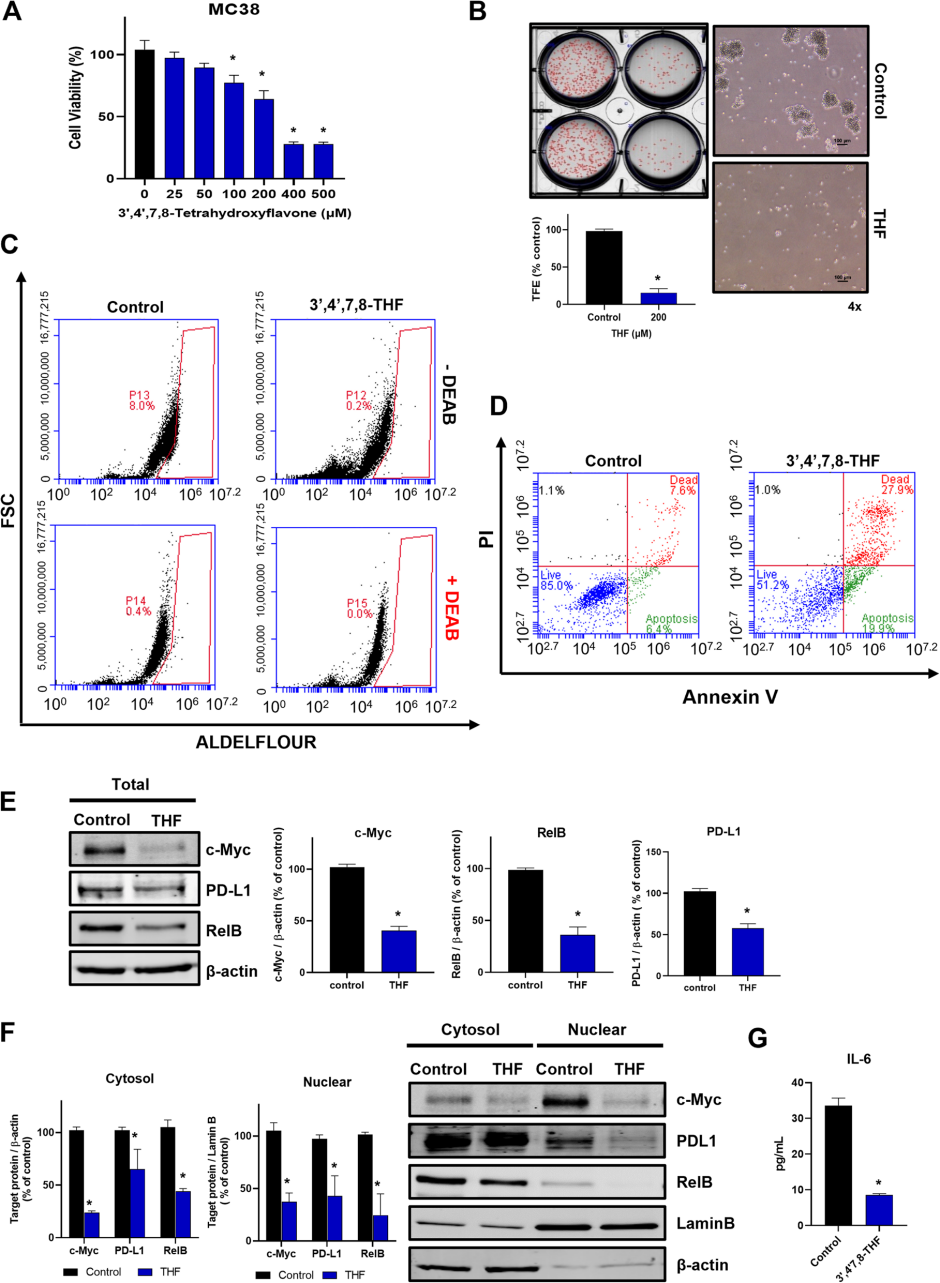
BRD4 inhibitory natural compound 3',4',7,8-tetrahydroxyflavone (THF) regulated the formation of tumorspheres derived from MC38 cells. **A** The inhibitory effect on the cell proliferation of THF in mouse colon cells (MC38 cells). The cells were cultured with the indicated increasing concentration range of THF for 24 h. Cell viability was measured using the MTS assay. **B** The inhibitory effect of tumorsphere formation by THF. Tumorspheres derived from MC38 cells were treated with 200 µM THF for 7 days. Tumorsphere images (right) were taken at ×10 magnification. **C** CSC marker, ALDH expression of MC38 cells. The cells were treated with 200 µM THF for 1 day. ALDH expression was measured using the ALDEFLUOR assay kit and a flow cytometer, as described in the “Materials and methods” section. **D** THF induced apoptosis in tumorspheres derived from MC38 cells. The tumorspheres were treated with 200 µM THF for 1 days. Apoptosis was analyzed using Annexin V/PI staining, as described in the “Material and methods” section. **E** Protein expression regulation of THF in tumorspheres. The protein expressions of c-Myc, PD-L1, and RelB were detected by Western blot. The cells were treated with 200 µM THF for 24 h. **F** Protein expression regulation of THF in the nuclear and cytosolic fractions of tumorspheres. The protein expressions of c-Myc, PD-L1, and RelB were detected by Western blot. The cells were treated with 200 µM THF for 24 h. **G** IL-6 level of tumorspheres under THF treatment. The level of IL-6 was examined in the THF (100 µM)-treated tumorspheres culture medium. The amount of IL-6 was quantified using a flow cytometer. Experiment values are represented as the mean ± SD of triplicates. Compared with the control as determined by student’s t-test or one-way ANOVA with Dunnett’s multiple comparisons tests, *p < 0.05

The MC-38 cell line derived from C57BL/6 murine colon adenocarcinoma cells can be employed as it has expressed PD-L1 and secretory IL-6 [38, 39]. We confirmed THF as an anti-CSC agent using MC-38 murine colon carcinoma cells. THF inhibits cell proliferation at 100 µM and tumorsphere formation at 200 µM (Fig. 8A, B). As ALDH1 is a colon CSC marker, we checked the ALDH1 levels of MC-38 under THF treatment. This compound reduced the ALDH1 activity of the MC-38 subpopulation and induced the apoptosis of tumorspheres derived from MC-38 colon cancer cells (Fig. 8C, D). To confirm the biochemical function of THF on MC-38 cells, we analyzed the levels of c-myc, PD-L1, RelB, and IL-6. Our data showed that THF reduced total protein levels of c-myc, PD-L1, and RelB and decreased nuclear protein levels of c-myc, PD-L1, and RelB on tumorspheres derived from MC-38 cells (Fig. 8E, F). As THF reduced secretory IL-6 levels (Fig. 8G), THF reduced colon CSC formation through the BRD4/PD-L1/RelB/IL-6 axis.

### Antitumor effect of THF using MC38 syngeneic xenograft model

**Fig. 9:**
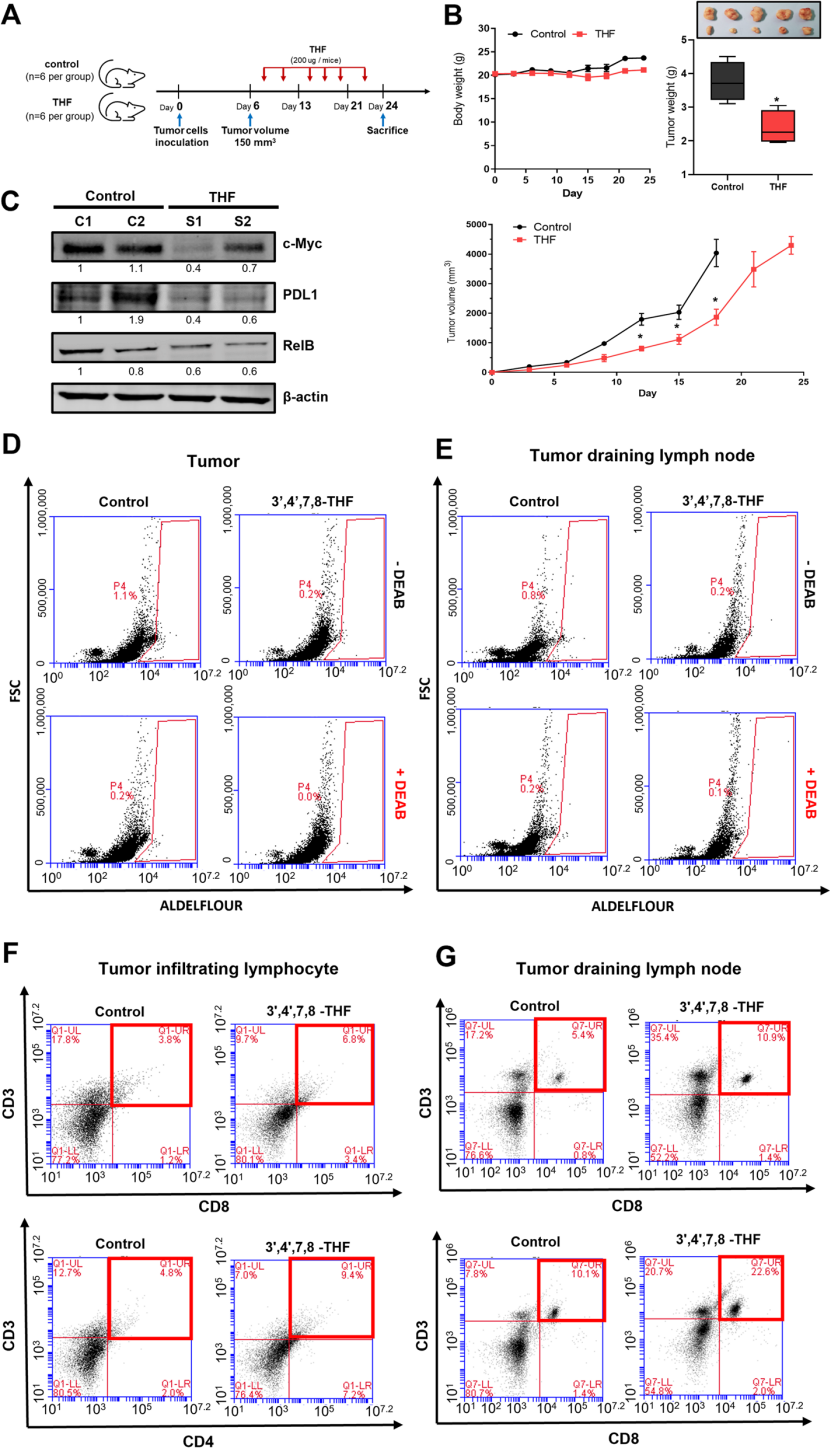
Effect of 3',4',7,8-tetrahydroxyflavone (THF) on tumor growth and immune response in mouse models. **A** In vivo experimental schedule. **B** Effect of THF of the mouse tumor. Mice were inoculated with MC38 cells and treated with THF. The body weight and tumor volumes of the mice were monitored for 25 days. After being sacrificed, tumor weight was determined. *n* = 5 in each group. **C** Protein expression regulation of THF in the tumor. The protein expressions of c-Myc, PD-L1, and RelB in tumors were detected by Western blot. **D, E** CSC marker, ALDH, expression of tumor or tumor-draining lymph nodes (TDLNs). Tumors or TDLNs were isolated into single cells, as described in the “Materials and methods” section. ALDH expression was measured using the ALDEFLUOR assay kit and a flow cytometer. **F**, **G** Helper T-cells or cytotoxic T-cells contained in the tumors and TDLNs. Tumors or TDLNs were isolated into single cells and analyzed using CD3^+^/CD4^+^ or CD3^+^/CD8^+^ staining, as described in the “Materials and methods” section. Compared with the control as determined by student’s t-test or one-way ANOVA with Dunnett’s multiple comparisons tests, *p < 0.05

The MC-38 cell line derived from C57BL/6 murine colon adenocarcinoma cells and 4T1 cell line from BALB/c murine breast cancer can be employed as a robust preclinical immuno-oncology model and expressed PD-L1 and secretory IL-6. 4T1 and MC-38 cells were used to evaluate methods of disrupting tumor-infiltrating lymphocyte (TIL) inhibitors and investigate methods of improving immune checkpoint blockade (ICB) therapy (such as PD-L1 blockade) [40]. As THF has an anti-proliferative effect on MC-38 cells, we used an in vivo mouse model to examine whether it reduces tumor growth (Fig. 9A and Fig. S5A). The body weights of the control and THF-treated C57BL/6 and BALB/c murine mice did not change (Fig. 9B and Fig. S5B). The weights and volume of tumors from THF-treated C57BL/6 and THF-treated BALB/c murine were lower and small than those of tumors from control C57BL/6 and BALB/c murine (Fig. 9B and Fig. S5B). To demonstrate the effect of THF on MC-38 and 4T1 tumor in vivo, we performed Western blotting of the resected tumor tissue. Reduced expressions of c-myc, PD-L1, and RelB proteins were observed in THF-treated MC38 tumor tissues compared with the control group (Fig. 9C and Fig. S5C). Our results indicated that THF effectively reduced tumor growth. We isolated 4T1-derived and MC38-derived tumor and TDLNs using Evans blue staining and checked the colon CSC marker, ALDH1, using the ALDEFLOUR™ assay. THF reduced the ALDH1-positive subpopulation from 2.4 to 1.6% in the MC-38 colon tumor and 1.1–0.2% in the 4T1 breast tumor (Fig. 9D and Fig. S5D) and the ALDH-expressing population from 2.2 to 0.2% and 0.8–0.2% in TDLN (Fig. 9E and Fig. S5D). These results showed that THF reduced the frequency of ALDH1-expressing subpopulations of tumor and TDLNs and the colon CSC trait. THF increased the levels of CD3^+^/CD4^+^ and CD3^+^/CD8^+^ T-cells in the tumor and TDLNs of the MC-38 and 4T1 tumor bearing mice (Fig. 9F, G and Fig.S5E).

## Discussion

CSCs have been isolated and established from human tumors and are the driving forces of tumor recurrence and metastasis [41, 42]. Targeting CSC self-renewal or stemness was known as an effective cancer therapy [43]. Previous reports have shown that combined inhibition of BRD4 and RAC1 suppresses growth, stemness, and tumorigenesis by disrupting the c-MYC/G9a/FTH1axis [44]. In this study, we examined the biological function of targeting BRD4 using siRNA, inhibitor, and degrader on breast CSCs. We showed that the siRNA for BRD4, a specific inhibitor of BET family proteins (JQ1), and BRD4 protein degrader (ARV-825) suppress mammosphere formation, ALDH1 activity, CD44^+^/CD24^−^ subpopulation, CSC growth, and specific survival factor of CSCs. The expression level of CSC BRD4 is higher than that in BCs (Figs. 1 and 2). BRD4 participates in breast CSC formation and the tumorigenic activity of MC38 derived from tumors (Figs. 1 and 9).

BRD4 can regulate PD-L1 expression in TNBC [45]. We examined the biological function of PD-L1 by targeting PD-L1 using siRNA, BRD4 inhibitors (JQ1 and ARV-825), and PD-L1 degrader (verteporfin) on breast CSCs. We found that siRNA for PD-L1, a specific inhibitor of BET family proteins (JQ1), and BRD4 protein degrader (ARV-825) suppress mammosphere formation. BRD4 inhibition reduced the expression levels of the membrane, cytoskeleton, and nuclear fraction (Fig. 3). For the first time, we showed that nuclear PD-L1 regulated mammosphere formation. PD-L1 or B7-H1 is well known for its role in immune checkpoint regulation as membrane function. Nuclear PD-L1 of cancer cells regulated the sister chromatid cohesion of BC and was independent of its function in the immune checkpoint [30]. Nuclear PD-L1 regulated gene expressions of NF-κB signaling (BIRC3, RelB, and TRAP1), MHC class I (HLA-A, HLA-B, and HLA-H), and immune checkpoint (PD-L2, VISTA, and B&-H3) [29]. NF-κB signaling is essential for breast CSC formation. We checked whether verteporfin (a PD-L1 degrader) can downregulate the transcript and protein levels of RelB. Verteporfin downregulated the transcript and protein levels of RelB (Fig. 4). The siRNA and inhibitor of RelB (calcitriol) suppressed mammosphere formation, and for the first time, we showed that RelB regulated mammosphere formation. The RelB/RelA (p65) complex promotes the transcript of the *NF-kB* target gene and *IL-6* gene of ER-negative BC [34]. RelB interacts with RelA (p65), and the RelB/RelA (p65) complex binds the promoter sequence of the *IL-6* gene (Fig. 6). A new finding is that the RelB protein regulates breast CSC formation through IL-6 regulation. Our results indicated that RelB may be a potential target of BC therapy.

Recently, studies on cytokine-modulated tumor microenvironments and BCSCs have focused on the mechanisms of chemoresistance [46]. IL-6 in the tumor microenvironment can regulate the self-renewal and survival of breast CSCs. Our data showed that the inhibitors of BRD4/PD-L1/RelB/p65 reduced the levels of secretory IL-6 in breast CSCs (Figs. 6 and 7). The combinational therapeutic strategy of targeting breast CSCs and neutralizing IL-6 may be a good chance to enhance the survival of patients with BC. Our experiment showed that BRD4/PD-L1/RelB/IL-6 axis regulated breast CSCs formation and it was confirmed by the rescue experiments using the PD-L1 overexpression plasmid (Fig. 6F).

Our experiment showed that the inhibition of the PD-L1 pathway results in anti-CSC and antitumor conditions. We used MC38 colorectal cancer cells and 4T1 breast cancer cells, which are sensitive to PD-L1 monotherapy and express PDL-1 and IL-6 [40]. We examined the relationship between PD-L1 and CSC formation on MC38 colorectal cancer, MC38-derived tumor, and 4T1-derived tumor using THF, a natural compound of BRD4 inhibitor. CSCs from MC38 colorectal cancer and MC38-derived tumors showed that THF inhibited CSC formation and induced apoptosis of MC38-derived CSCs through the c-Myc/BRD4/PD-L1/RelB/IL6 axis (Fig. 8). THF reduced the MC38-derived and 4T1-derived tumor volume and weight, and the inhibitor-treated tumor showed reduced expression levels of c-Myc, PD-L1, and RelB. The strategy of targeting BC and breast CSCs using a natural BRD4 inhibitor may help promote the survival of patients with BC.

PD-L1 on MC38 colorectal adenocarcinoma cells and 4T1 breast cancer cells is sufficient to suppress antitumor immunity through tumor immune evasion or correlates with an inflamed tumor microenvironment and inhibits CD8 T-cell cytotoxicity. To evaluate the significance of PD-L1 and BRD4 inhibitor on tumors, we used a mouse tumor model sensitive to PD-1 blockade [40]. To examine the in vivo treatment effect of THF, we checked the levels of CD3 + CD4 + and CD3 + CD8 + T-cells in TIL and TDLNs. THF increased the levels of CD3 + CD4 + and CD3 + CD8 + T-cells in TIL and TDLNs. ICB (anti-PD-L1) and BRD4 inhibitor THF showed a similar anticancer effect.

## Conclusion

**Fig. 10:**
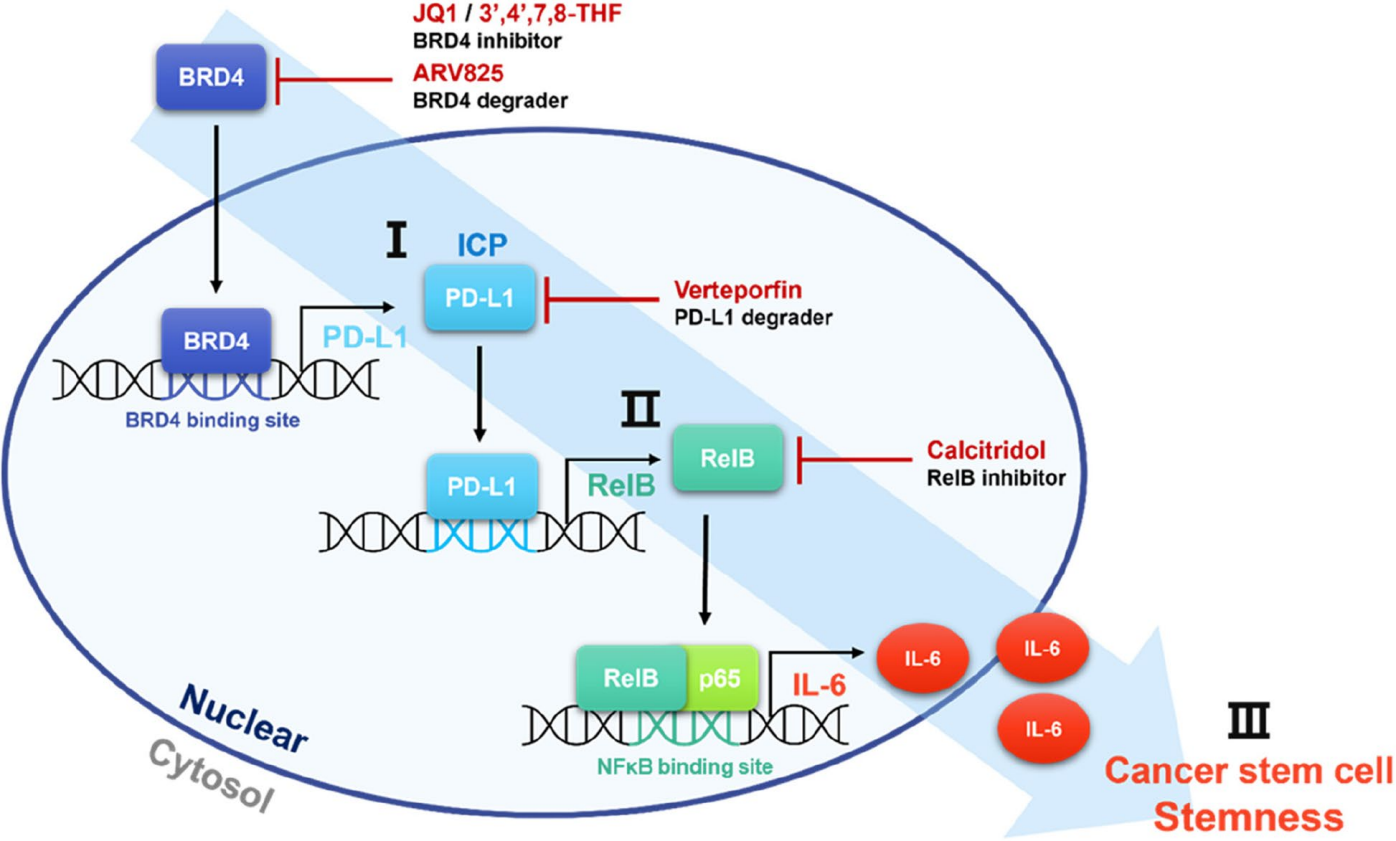
Proposed model for cancer stem cell formation and immune response by BRD4/PD-L1/RelB/IL-6. The BRD4 protein transcriptionally regulated the *PD-L1* gene, and PD-L1 protein transcriptionally regulated the *RelB* gene. The RelB/p65 complex regulates IL-6 transcripts. The BRD4/PD-L1/RelB/IL-6 axis regulated breast CSC formation

Our findings established a strong connection between BRD4/PD-L1/RelB/IL-6 and CSC stemness in BC. By providing unidentified evidence that nuclear PD-L1 and RelB promote IL-6 production, the BRD4 inhibitor repressed breast CSC formation, suppressed immune evasion in immunogenic tumors, and increased CD8 + T-cell cytotoxicity (Fig. 10).

### Supplementary Information

**Additional file 1: Fig. S1.** Change of sister chromatid by programmed death-ligand 1 (PD-L1) depletion on MDA-MB-231 cells. Chromosome spread assay using MDA-MB-231 cells to analyze sister chromatid. (A) The cells were treated with 2 µM verteporfin or (B) siRNA of PD-L1. After 1 day, the cells were incubated with 10 µM/mL colcemid (Thermo Fisher Scientific Inc., Waltham, MA, USA) for 2 h at 37℃. Miotic cells were incubated with RPMI: water (2:3) solution for 6 min at RT and fixed with methanol: acetic acid (3:1) solution. Fixed cells stained with 5% Giemsa solution (Thermo Fisher Scientific Inc., Waltham, MA, USA) in glass slides. **Fig. S2.** Promoter sequences of programmed death-ligand 1 containing the BRD4-binding site and primer site. Immunoprecipitated DNA was amplified by qPCR using primers specific and primer sequences represented by blue. Red sequences indicate BRD4 binding region. **Fig. S3.** Promoter sequences of interleukin-6 containing the NF-kB binding site and primers sites. Immunoprecipitated DNA was amplified by qPCR using primers specific and primer sequences represented by blue. Red sequences indicate NF-kB binding region. **Fig. S4.** CSC marker expressions in MDA-MB-231 cells treated with JQ1. The cells were treated with 1 µM JQ1 for 18 h. The expression levels of OCT4 and SOX2 were measured by western blot. Internal control of total fraction was used as β-actin. **Fig. S5.** Effect of 3',4',7,8-tetrahydroxyflavone (THF) on tumor growth and immune response in 4T1 mouse models. A In vivo experimental schedule. B Effect of THF of the mouse tumor. Mice were inoculated with 4T1 cells and treated with THF. The body weight and tumor volumes of the mice were monitored for 25 days. After being sacrificed, tumor weight was determined. *n* = 6 in each group. C Protein expression regulation in tumors of THF-untreated and THF-treated mice. The protein expressions of c-Myc, PD-L1, and RelB in tumors were detected by Western blot. D CSC marker, ALDH expression of the tumor. Tumors and TDLNs were isolated into single cells, as described in the “Materials and methods” section. ALDH expression was measured using the ALDEFLUOR assay kit and a flow cytometer. E Helper T-cells or cytotoxic T-cells contained in the tumors and TDLNs. Tumors or TDLNs were isolated into single cells and analyzed using CD3+/CD4 + or CD3+/CD8 + staining, as described in the “Materials and methods” section. Compared with the control as determined by student’s t-test or one-way ANOVA with Dunnett’s multiple comparisons tests, *p < 0.05.

## Data Availability

The datasets used for the current study are available corresponding author on reasonable request.

## Abbreviations

BC: Breast cancer
CSCs: Cancer stem cells
BCSC: Breast cancer stem cell
TNBC: Triple-negative breast cancer
MFE: Mammosphere formation efficiency
TFE: Tumorsphere formation efficiency
CUT&RUN: Cleavage Under Targets & Release Using Nuclease
BET: Bromodomain and extra terminal domain
CAPE: Caffeic acid phenethyl ester
THF: 3',4',7,8-Tetrahydroxyflavone
BRD4: Bromodomain-containing protein 4
CD274: Cluster of differentiation 274
PD-L1: Programmed death-ligand 1
NF-κB: Nuclear factor-kappa B
ChIP: Chromatin immunoprecipitation
IL-6: Interleukin-6
IL-8: Interleukin-8
TIL: Tumor-infiltrating lymphocyte
TDLN: Tumor-drained lymph node
