# Supplementary material for: BRD4/nuclear PD-L1/RelB circuit is involved in the stemness of breast cancer cells
Source: Cell Commun Signal. 2023 Nov 3;21:315. doi: 10.1186/s12964-023-01319-6 (PMC10623882; doi:10.1186/s12964-023-01319-6)
Supplement: Supplementary file 2 — Additional file 1: Fig. S1. Change of sister chromatid by programmed death-ligand 1 (PD-L1) depletion on MDA-MB-231 cells. Chromosome spread assay using MDA-MB-231 cells to analyze sister chromatid. (A) The cells were treated with 2 µM verteporfin or (B) siRNA of PD-L1. After 1 day, the cells were incubated with 10 µM/mL colcemid (Thermo Fisher Scientific Inc., Waltham, MA, USA) for 2 h at 37℃. Miotic cells were incubated with RPMI: water (2:3) solution for 6 min at RT and fixed with methanol: acetic acid (3:1) solution. Fixed cells stained with 5% Giemsa solution (Thermo Fisher Scientific Inc., Waltham, MA, USA) in glass slides. Fig. S2. Promoter sequences of programmed death-ligand 1 containing the BRD4-binding site and primer site. Immunoprecipitated DNA was amplified by qPCR using primers specific and primer sequences represented by blue. Red sequences indicate BRD4 binding region. Fig. S3. Promoter sequences of interleukin-6 containing the NF-kB binding site and primers sites. Immunoprecipitated DNA was amplified by qPCR using primers specific and primer sequences represented by blue. Red sequences indicate NF-kB binding region. Fig. S4. CSC marker expressions in MDA-MB-231 cells treated with JQ1. The cells were treated with 1 µM JQ1 for 18 h. The expression levels of OCT4 and SOX2 were measured by western blot. Internal control of total fraction was used as β-actin. Fig. S5. Effect of 3',4',7,8-tetrahydroxyflavone (THF) on tumor growth and immune response in 4T1 mouse models. A In vivo experimental schedule. B Effect of THF of the mouse tumor. Mice were inoculated with 4T1 cells and treated with THF. The body weight and tumor volumes of the mice were monitored for 25 days. After being sacrificed, tumor weight was determined. n = 6 in each group. C Protein expression regulation in tumors of THF-untreated and THF-treated mice. The protein expressions of c-Myc, PD-L1, and RelB in tumors were detected by Western blot. D CSC marker, ALDH expression of the tumor. Tu [file 12964_2023_1319_MOESM1_ESM.pdf]

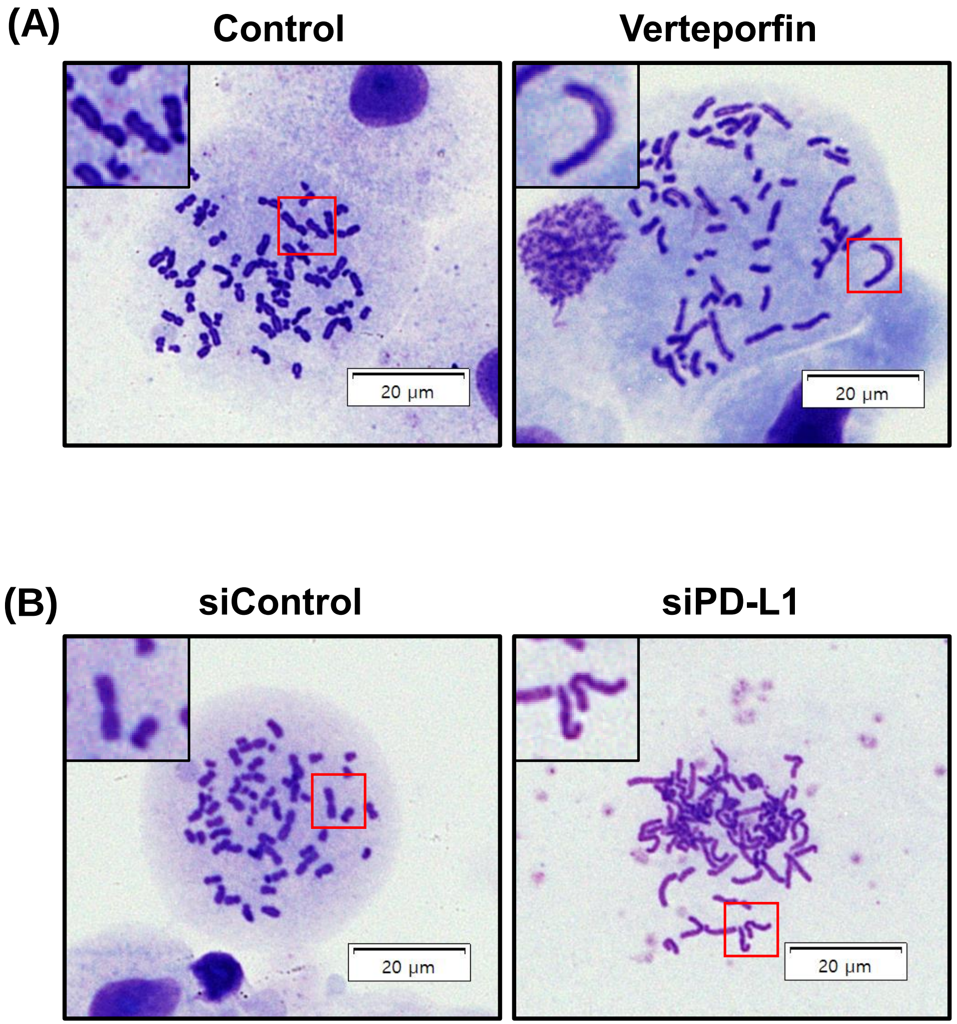

Fig. S1. Change of sister chromatid by programmed death-ligand 1 (PD-L1) depletion on MDA-MB-231 cells. Chromosome spread assay using MDA-MB-231 cells to analyze sister chromatid. (A) The cells were treated with 2 μM verteporfin or (B) siRNA of PD-L1. After 1 day, the cells were incubated with 10 μM/mL colcemide (Thermo Fisher Scientific Inc., Waltham, MA, USA) for 2 h at 37℃. Miotic cells were incubated with RPMI: water (2:3) solution for 6 min at RT and fixed with methanol: acetic acid (3:1) solution. Fixed cells stained with 5% Giemsa solution (Thermo Fisher Scientific Inc., Waltham, MA, USA) in glass slides.

AGGGCTTTCTTAACCCTCACCTAGAATAGGCTTCCGCAGCCTTAATCCTTAGGGTGGCAGAATATCAGGGACCCTGAGCATTCTTAAA

-1607 **-1588**

AGATGTAGCTCGGGATGGGAAGTTCTTTT**AATGACAAAGCAAATGAAG**TTTCATTATGTCGAGGAACTTTGAGGAAGTCACAGAAT

Primer F

-1515 **-1500**

CCACGATTTAAAAATATATTTCCTATTATACACCCA**TACACACACACACAC**ACCTACTTTCTAGAATAAAAACCAAAGCCATATGGGT

BRD4 binding site

-1417 **-1433**

CTGCTGCTGACTTTTTATATGTTGTAGAGTTATATCAAGTTATGTCAA**GATGTTCAGTCACCTT**GAAGAGGCTTTTATCAGAAAGGGG

Primer R

GACGCCTTTCTGATAAAGGTTAAGGGGTAACCTTAAGCTCTTACCCCTCTGAAGGTAAAATCAAGGTGCGTTCAGATGTTGGCTTG

Fig. S2. Promoter sequences of programmed death-ligand 1 containing the BRD4-binding site and primer site. Immunoprecipitated DNA was amplified by qPCR using primers specific and primer sequences represented by blue. Red sequences indicates BRD4 binding region.

-144
GCTGCACTTTTCCCCCTAGTTGTGTCTTGCGATGCTAAAGGACGTCACATTGCACAATCT**TAATAAGGTTTC**

-124 -73 -63
CAATCAGCCCCACCCGCTCTGGCCCCACCCTCACCCTCCAACAAAGATTTATCAAATGT**GGGATTTTCC**CAT
Primer F NFkB binding site

-31 -10
GAGTCTCAATATTAGAGTCTCAACCCCC**AATAAATATAGGACTGGAGAT**GTCTGAGGC**T**CATTCTGCCCTCG
Primer R TSS

AGCCCACCGGGAACGAAAGAGAAGCTCTATCTCCCCTCCAGGAGCCCAGCTATGAACTCCTTCTCCACAAG

Fig. S3. Promoter sequences of interleukin-6 containing the NF-kB binding site and primers sites
Immunoprecipitated DNA was amplified by qPCR using primers specific and primer sequences represented by blue. Red sequences indicate NF-kB binding region.

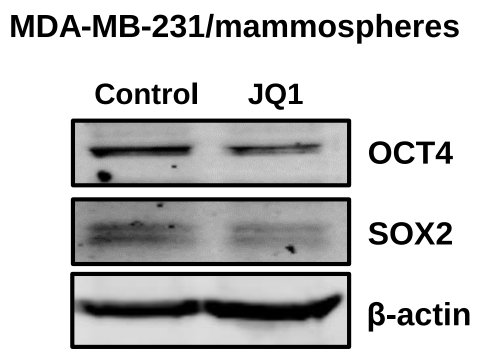

Fig. S4. CSC marker expressions in MDA-MB-231 cells treated with JQ1. The cells were treated with 1 μM JQ1 for 18 h. The expression levels of OCT4 and SOX2 were measured by western blot**.** Internal control of total fraction was used as β-actin.

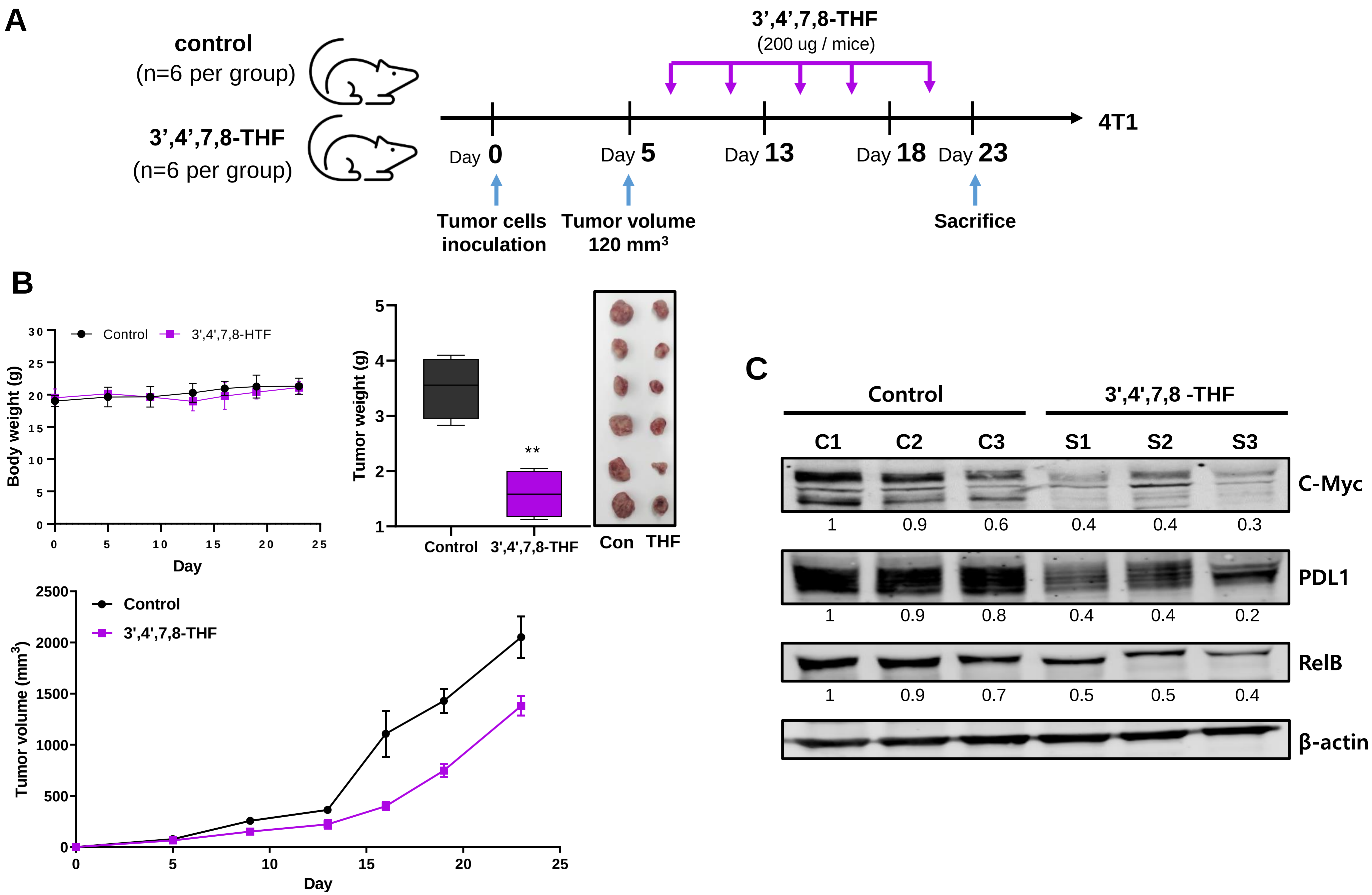
A
control
(n=6 per group)
3',4',7,8-THF
(n=6 per group)
3',4',7,8-THF
(200 ug / mice)
4T1
Day 0
Day 5
Day 13
Day 18
Day 23
Tumor cells inoculation
Tumor volume 120 mm3
Sacrifice
B
Control
3',4',7,8-HTF
Body weight (g)
Day
Tumor weight (g)
**
Control
3',4',7,8-THF
Con
THF
Control
3',4',7,8-THF
Tumor volume (mm3)
Day
C
Control
3',4',7,8 -THF
C1
C2
C3
S1
S2
S3
C-Myc
1 0.9 0.6 0.4 0.4 0.3
PDL1
1 0.9 0.8 0.4 0.4 0.2
RelB
1 0.9 0.7 0.5 0.5 0.4
β-actin

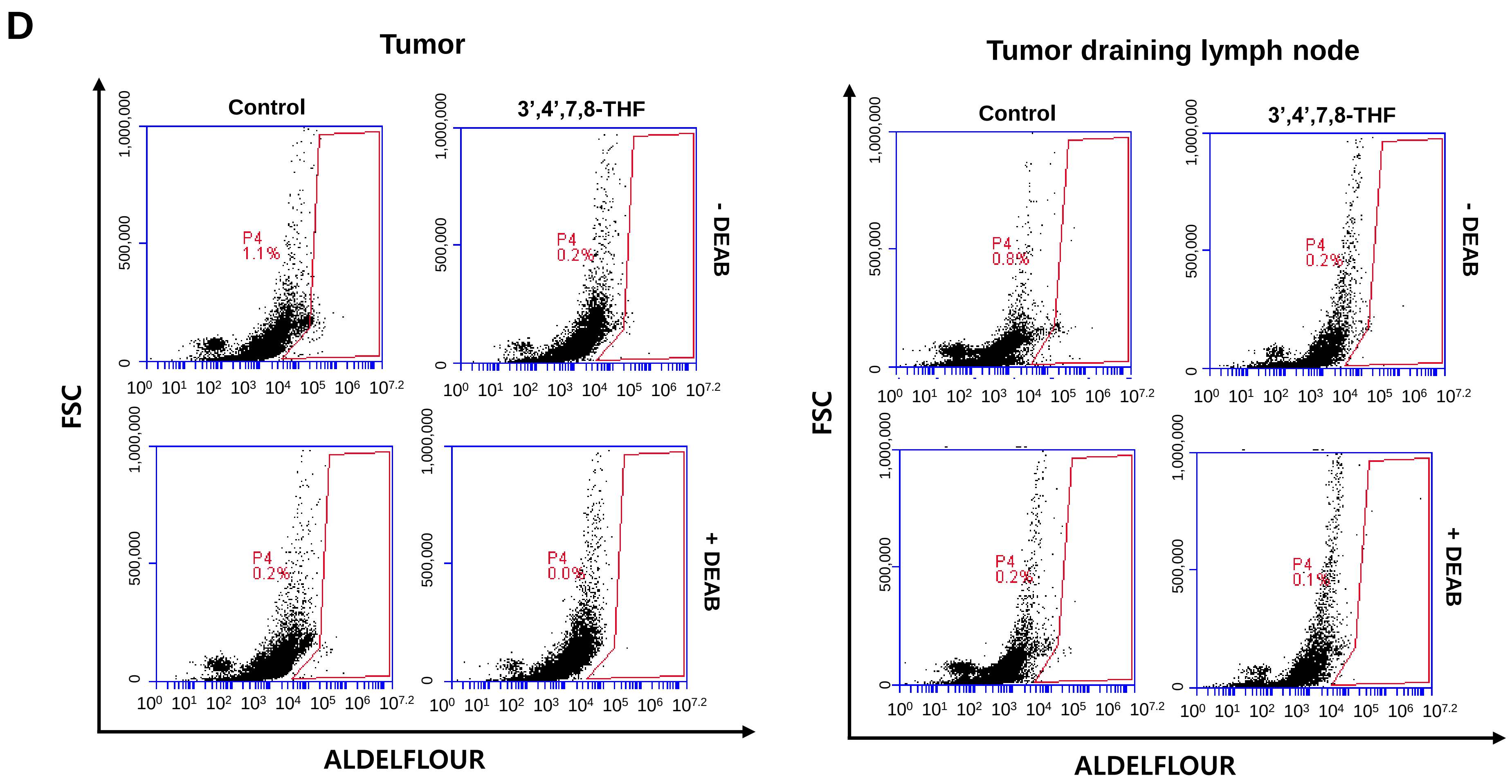
D
Tumor
Tumor draining lymph node
Control
3',4',7,8-THF
- DEAB
+ DEAB
P4 1.1%
P4 0.2%
P4 0.2%
P4 0.0%
P4 0.8%
P4 0.2%
P4 0.2%
P4 0.1%
FSC
ALDELFLOUR

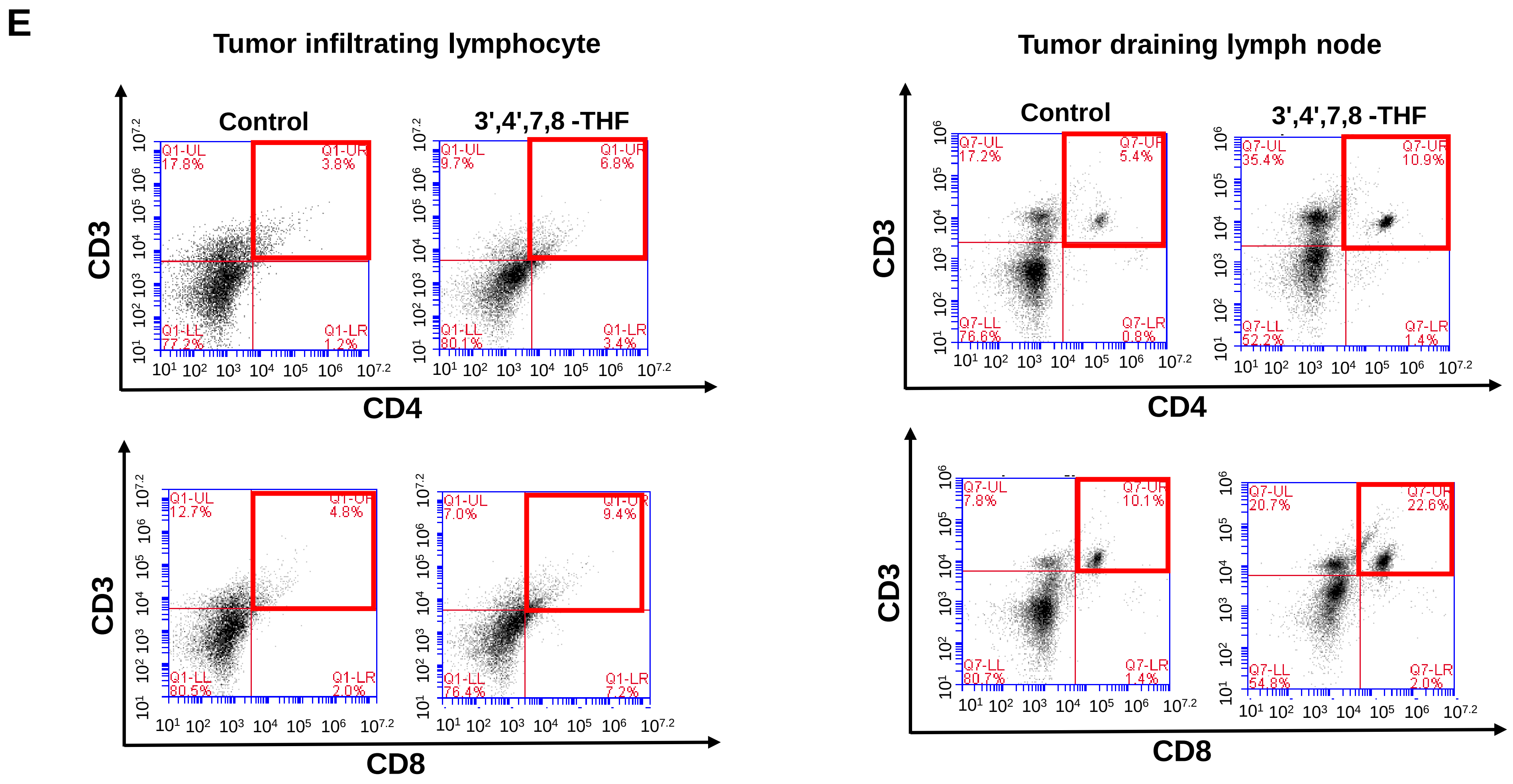

Fig. S5. Effect of 3',4',7,8-tetrahydroxyflavone (THF) on tumor growth and immune response in 4T1 mouse models**. A** In vivo experimental schedule. **B** Effect of THF of the mouse tumor. Mice were inoculated with 4T1 cells and treated with THF. The body weight and tumor volumes of the mice were monitored for 25 days. After being sacrificed, tumor weight was determined. n = 6 in each group. **C** Protein expression regulation in tumors of THF-untreated and THF-treated mice. The protein expressions of c-Myc, PD-L1, and RelB in tumors were detected by Western blot. **D** CSC marker, ALDH expression of the tumor. Tumors and TDLNs were isolated into single cells, as described in the Materials and Methods. ALDH expression was measured using the ALDEFLUOR assay kit and a flow cytometer. **E** Helper T-cells or cytotoxic T-cells contained in the tumors and TDLNs. Tumors or TDLNs were isolated into single cells and analyzed using $CD3^+/CD4^+$ or $CD3^+/CD8^+$ staining, as described in the Materials and Methods. Compared with the control as determined by student's t-test or one-way ANOVA with Dunnett's multiple comparisons tests, *$p < 0.05$.
